# Traditional Aboriginal Preparation Alters the Chemical Profile of *Carica papaya* Leaves and Impacts on Cytotoxicity towards Human Squamous Cell Carcinoma

**DOI:** 10.1371/journal.pone.0147956

**Published:** 2016-02-01

**Authors:** Thao T. Nguyen, Marie-Odile Parat, Paul N. Shaw, Amitha K. Hewavitharana, Mark P. Hodson

**Affiliations:** 1 School of Pharmacy, The University of Queensland, Brisbane, Queensland, Australia; 2 Metabolomics Australia, Australian Institute for Bioengineering and Nanotechnology, The University of Queensland, Brisbane, Queensland, Australia; University of Alabama at Birmingham, UNITED STATES

## Abstract

*Carica papaya* leaf decoction, an Australian Aboriginal remedy, has been used widely for its healing capabilities against cancer, with numerous anecdotal reports. In this study we investigated its *in vitro* cytotoxicity on human squamous cell carcinoma cells followed by metabolomic profiling of *Carica papaya* leaf decoction and leaf juice/brewed leaf juice to determine the effects imparted by the long heating process typical of the Aboriginal remedy preparation. MTT assay results showed that in comparison with the decoction, the leaf juice not only exhibited a stronger cytotoxic effect on SCC25 cancer cells, but also produced a significant cancer-selective effect as shown by tests on non-cancerous human keratinocyte HaCaT cells. Furthermore, evidence from testing brewed leaf juice on these two cell lines suggested that the brewing process markedly reduced the selective effect of *Carica papaya* leaf on SCC25 cancer cells. To tentatively identify the compounds that contribute to the distinct selective anticancer activity of leaf juice, an untargeted metabolomic approach employing Ultra High Performance Liquid Chromatography-Quadrupole Time of Flight-Mass Spectrometry followed by multivariate data analysis was applied. Some 90 and 104 peaks in positive and negative mode respectively were selected as discriminatory features from the chemical profile of leaf juice and >1500 putative compound IDs were obtained *via* database searching. Direct comparison of chromatographic and tandem mass spectral data to available reference compounds confirmed one feature as a match with its proposed authentic standard, namely pheophorbide A. However, despite pheophorbide A exhibiting cytotoxic activity on SCC25 cancer cells, it did not prove to be the compound contributing principally to the selective activity of leaf juice. With promising results suggesting stronger and more selective anticancer effects when compared to the Aboriginal remedy, *Carica papaya* leaf juice warrants further study to explore its activity on other cancer cell lines, as well as investigation to confirm the identity of compounds contributing to its selective effect, particularly those compounds altered by the long heating process applied during the traditional Aboriginal remedy preparation.

## Introduction

*Carica papaya* is a fast growing, soft-wooded, herbaceous plant reaching 3–10 m in height. The leaves are large (30–60 cm long), yellow-green to dark-green in colour, palmately-lobed, arranged spirally and clustered at the crown. In traditional medicine, *Carica papaya* leaves have been used for treatment of asthma, colic, fever, beriberi (thiamine deficiency), and as an abortifacient in India [1], for malaria and dengue fever in Sri Lanka, Pakistan, Malaysia [2–4] and cancer in Vietnam and Australia [5–7].

*Carica papaya* leaf decoction, an Australian Aboriginal remedy, has been reported widely for its healing capabilities against cancer, with numerous anecdotal reports [8]. Recently, scientific studies have demonstrated the inhibitory activity of this decoction on the proliferation of both haematopoietic cell lines and solid tumour cell lines [9, 10]. The decoction was prepared by brewing *Carica papaya* leaves with water in a glass beaker or wooden vessel for a period of time (varying from 5 minutes to 12 hours in various reports). The heating process to prepare the decoction can potentially affect temperature-sensitive compounds, leading to possible changes in bioactivity. This type of effect has been reported for other herbal medicines such as ginseng root and ginger, where steaming preparation has been observed to increase the anti-proliferative effect compared to unprocessed materials [11–13]. We therefore conducted a study to test the hypothesis that the preparation of *Carica papaya* leaves following an Aboriginal remedy alters the chemical pattern and thus the anticancer effect of *Carica papaya* leaf.

Human squamous cell carcinoma SCC25 cells and non-cancerous human HaCaT keratinocytes were selected for the cytotoxic studies of papaya leaf because skin and oral mucosa are directly accessible to the tested extracts, *i*.*e*. they do not require gastrointestinal absorption for the cells to be exposed to bioactive compounds. Moreover, squamous cell carcinoma, together with basal cell carcinoma, are the most frequent environmentally induced cancers, with UV light being recognized as a key etiologic factor [14–18], which is a significant issue in Australia. Research into the development of new therapeutic agents is of considerable importance and attention has recently focused on natural products. Interestingly, there is some overlap between the geographical distribution of *Carica papaya* [19] and that of UV exposure [20]. As the widespread use of different parts of *Carica papaya* plants for the treatment of skin infections and wound healing has been scientifically validated [21–23], testing of the effect of papaya leaf preparations on skin cancer is warranted.

With the application of an untargeted metabolomic approach employing Ultra High Performance Liquid Chromatography-Quadrupole Time of Flight-Mass Spectrometry followed by multivariate data analyses, our study aimed to explore the relationship of the heating process with the chemical constituents and biological activity to tentatively identify the bioactive compounds responsible for the difference in the anticancer effect of the extracts.

## Materials and Methods

Fresh *Carica papaya* leaves were collected from Tropical Fruit World (TFW), a privately-owned plantation orchard farm and research park in northern New South Wales, Australia (http://www.tropicalfruitworld.com.au/). Permission for the use of the *Carica papaya* leaves was granted by Aymon Gow, manager of TFW. The papaya plants in this facility are neither protected nor endangered species.

### Chemicals and reagents

Dulbecco’s Modified Eagle’s Medium (DMEM), DMEM-F12, penicillin/streptomycin, trypsin, fetal bovine serum (FBS) were purchased from Invitrogen (Life Technologies, Mulgrave, VIC, Australia). Dihydrocoumarin (2-chromanone; 99% purity), fucoxanthin ((3*S*,3'*S*,5*R*,5'*R*,6*S*,6'*R*,8'*R*)-3,5'-dihydroxy-8-oxo-6',7'-didehydro-5,5',6,6',7,8-hexahydro-5,6-epoxy-β,β-caroten-3'-yl acetate; 99% purity), pheophorbide A (3-[(3*S*,4*S*,21*R*)-14-ethyl-21-(methoxycarbonyl)-4,8,13,18-tetramethyl-20-oxo-9-vinyl-3-phorbinyl]propanoic acid; 99% purity), 3-(4,5-dimethylthiazol-2-yl)-2,5-diphenyltetrazolium bromide (MTT), dimethyl sulfoxide (DMSO), LC-MS grade ammonium bicarbonate and formic acid were obtained from Sigma-Aldrich (Castle Hill, NSW, Australia). Anacardic acid (2-hydroxy-6-pentadecylbenzoic acid; >98% purity), cinnamic acid ((2*E*)-3-phenylacrylic acid; >95% purity), dihydromethysticin (6-[2-(1,3-benzodioxol-5-yl)ethyl]-4-methoxy-5,6-dihydro-2H-pyran-2-on; >98% purity), fucoxanthin ((3*S*,3'*S*,5*R*,5'*R*,6*S*,6'*R*,8'*R*)-3,5'-dihydroxy-8-oxo-6',7'-didehydro-5,5',6,6',7,8-hexahydro-5,6-epoxy-β,β-caroten-3'-yl acetate; 99% purity), hyperforin ((1*R*,5*S*,6*R*,7*S*)-4-hydroxy-5-isobutyryl-6-methyl-1,3,7-tris(3-methyl-2-buten-1-yl)-6-(4-methyl-3-penten-1-yl)bicyclo[3.3.1]non-3-ene-2,9-dione; >90% purity), pinolenic acid ((5*Z*,9*Z*,1*2Z*)-5,9,12-octadecatrienoic acid; >98% purity), stearidonic acid ((6*Z*,9*Z*,12*Z*,15*Z*)-6,9,12,15-octadecatetraenoic acid; >98% purity), and traumatic acid ((2*E*)-2-dodecenedioic acid; >98% purity) were purchased from Sapphire Bioscience (Redfern, NSW, Australia). HPLC grade acetonitrile was obtained from Merck (Darmstadt, Germany). Purified water was generated using a Milli-Q system (Millipore, USA).

### Extract preparation

Fresh *Carica papaya* leaves were collected from Tropical Fruit World. The papaya plants in this facility had not been sprayed with any chemicals. The leaves were thoroughly washed under running tap water to remove any particulate matter and rinsed carefully with MilliQ water to obtain clean leaves.

#### Leaf decoction

Leaf decoction was prepared following the Australian remedy for cancer treatment; this recipe was also used in the recent investigations of Morimoto *et al*. [9] and Otsuki *et al*. [10]. The cleaned leaves (200 g) were partly dried in air at room temperature and then added to 2.5 L of deionized water in a glass beaker, brought to the boil and further heated uncovered for 4 hr. The leaf decoction was then lyophilised at -60°C and 0.1 mbar using a Christ Alpha 2-4LD freeze-dryer (Martin Christ Gefriertrocknungsanlagen GmbH, Germany) to obtain a brown leaf decoction powder, which was portioned for storage at -80°C prior to analysis.

#### Leaf juice

The method to obtain leaf juice was adapted from procedures used in studies for dengue fever (3, 4) or toxicity of *Carica papaya* leaves [24–26] as follows: The cleaned leaves were crushed using a mortar and pestle, without the addition of water. The juice was manually separated from the crushed leaf debris using clean muslin fabric, then lyophilised under the same conditions as the leaf decoction to obtain a dark green powder, which was portioned for storage at -80°C prior to analysis.

#### Brewed leaf juice

To comprehensively evaluate the effect of the heating process, we included in our study an extract obtained by brewing the leaf juice powder under the same condition as processing the whole leaf to obtain the leaf decoction. Leaf juice powder was added to deionised water in a glass beaker with a ratio 200 mg in 100 mL of water and heated to the boil then further heated uncovered for 4 hr to create brewed leaf juice. The brewed leaf juice was then lyophilised under the same conditions as the leaf decoction to obtain a brown brewed leaf juice powder, which was portioned for storage at -80°C prior to analysis.

#### Sample preparation for cytotoxic studies

Leaf juice powder, brewed leaf juice powder, and leaf decoction powder were dissolved at concentrations of 2 mg/mL in medium (stock solution). The stock solutions were sterile filtered through a 0.22 μm polyvinylidene fluoride (PVDF) sterile filter (Merck Millipore, Germany) and diluted to the desired final concentrations with medium before the test.

#### Sample preparation for chemical profile analysis by LC-MS

Leaf juice powder, brewed leaf juice powder, and leaf decoction powder were dissolved at concentrations of 2 mg/mL in water then the obtained solutions subsequently filtered through a 0.22 μm PVDF filter (Merck Millipore, Germany). When filtering, an adequate volume was passed through membrane filter; the first 1.0 mL was discarded and the remaining volume was collected in an HPLC vial, ready for injection.

To facilitate direct comparison among the extracts, the concentrations of extracts used in following cell viability assay and mass spectrometric/chemometric analysis were equivalently converted and expressed as concentrations of original leaf.

### Cell culture conditions

SCC25 cells (ATCC CRL-1628) were maintained in DMEM/F12 medium supplemented with 10% v/v heat-inactivated fetal bovine serum, 1% penicillin-streptomycin and 0.4 μg/mL hydrocortisone. HaCaT cells (a generous gift from Professor Fusenig) [27] were propagated in DMEM medium supplemented with 10% fetal bovine serum and 1% penicillin-streptomycin. The cells were grown in a humidified incubator at 37°C in a 5% CO_2_ atmosphere and passaged every 3 days. Experiments were performed once cultures were allowed to reach 70–90% confluence.

### Cell viability assay

The colorimetric tetrazolium dye procedure commonly referred to as the 3-(4,5-dimethylthiazol-2-yl)−2,5-diphenyltetrazolium bromide (MTT) assay, developed by Mosmann [28], was used (with minor modifications) to evaluate the effect of extracts on SCC25 and HaCaT viability. SCC25 or HaCaT cells were plated into 96-well plates at densities of 6 × 10^3^ cells per well in 100 μL of DMEM/F12 (10% serum) and 3 × 10^3^ cells well in 100 μL of DMEM (10% serum), respectively. Cells were incubated at 37°C for 24 hr and were subsequently treated for 48 hr with 0.5% serum medium containing increasing concentrations of extracts. Control cells were exposed to medium only. Cells were then incubated in 100 μL of MTT-containing medium (0.2 mg/mL MTT in 0.5% serum medium) at 37°C for an additional 2 hr. The medium was then removed and the formazan crystals trapped in cells were dissolved in 100 μL of DMSO by gentle shaking for 20 min on an orbital shaker. Absorbance of the solubilised product was measured at 595 nm using an Imark plate reader (BioRad, USA). The absorbance of cells exposed to medium only was taken as 100% cell viability (*i*.*e*. the control). The results were expressed as a percentage of the viability of control cells ± standard error of the mean (SEM) from 4–8 parallel determinations in three independent experiments (n = 3). Dose-effect analysis on SCC25 cells was performed by one-way analysis of variance (ANOVA) followed by Dunnett’s test. Differences between the SCC25 and HaCaT cell lines, and interaction between cell line and extract effects were analysed by two-way ANOVA with Bonferroni post-tests. All univariate statistical analyses were carried out using GraphPad Prism 6.0 (GraphPad Software Inc., San Diego, USA).

### UHPLC-ToF-MS analysis

Chromatographic analysis of compounds in papaya leaf extracts was performed on an Agilent 1290 UHPLC system (Agilent Technologies, Santa Clara, USA). Chromatographic separation was achieved using a 2.1×150 mm, 3.5 μm ECLIPSE PLUS C18 analytical column (Agilent) with guard protection. Mobile phase A was MilliQ water containing 5 mM ammonium bicarbonate and 0.1% formic acid, adjusted to pH 7.0 ± 0.1 and mobile phase B was 95% acetonitrile and 5% MilliQ water (*v/v*) containing 5 mM ammonium bicarbonate and 0.1% formic acid. The following gradient elution was adopted: 10 to 80% B from 0 to 42 min; 80 to 90% B from 42 to 45 min; 90 to 100% B from 45 to 46 min; held at 100% B from 46 to 48 min; returned to 10% B over the next 2 min; column re-equilibration with 10% B for 10 min prior to the next injection. Thus the total chromatographic run time was 60 min. A flow rate of 0.2 mL/min was applied and 20 μL of sample was injected.

Each extract was run in triplicate in both positive and negative ionisation mode. Mass spectrometric detection was performed on an Agilent 6520 high resolution accurate mass quadrupole time-of-flight (Q-ToF) mass spectrometer equipped with a multimode source operating in both Electrospray Ionisation (ESI) and Atmospheric Pressure Chemical Ionisation (APCI) modes. Mass spectral acquisition was controlled using MassHunter software (B.02.01 SP3—Agilent). The mass spectrometer was operated using a mass range of *m/z* 100–1700, at a scan rate of 3.0 cycles/second using the following acquisition parameters: capillary voltage 2500 V, nebulizer pressure 30 psi, drying gas flow 5.0 L/min, gas temperature 300°C, fragmenting voltage 175 V, skimmer voltage 65 V. To ensure the desired mass accuracy of recorded ions, continuous internal calibration was performed during analysis with the use of reference ions—*m/z* 121.050873 (protonated purine) and *m/z* 922.009798 (protonated hexakis) in positive mode; in negative mode, ions with *m/z* 119.0362 (deprotonated purine) and *m/z* 966.000725 (formate adduct of hexakis) were used to correct for scan to scan variations.

### MS data analysis

Data processing and analysis were performed using Agilent MassHunter Qualitative software (Version B.05.00) with Molecular Feature Extractor (MFE) algorithms in concert with Mass Profiler Professional software (Version 12.1, Agilent) to align features from the chromatograms of all nine samples from three extracts of papaya leaves. The following cut-off settings were employed: minimum peak filters of 500 counts, peak spacing tolerance of 0.0025 m/z plus 7.0 ppm, assigned charge states limited to a maximum of two, minimum compound filters of 3000 counts.

### Chemometric analysis

The data matrix obtained from MS data analysis was imported into SIMCA-P software (Version 13.0.3.0, MKS Umetrics AB, Sweden). Data were log_10_ transformed and mean centered prior to multivariate data analysis using principal component analysis (PCA—unsupervised analysis) and Orthogonal Projection to Latent Structures-Discriminant Analysis (OPLS-DA—supervised analysis). The scores and loadings plot from these analyses, which describe the multivariate relationships of the observations (samples) and the variables (LC-MS features) respectively, were then used to determine the features that contribute to the differences among the extracts.

Preliminary compound identification of the features of interest was performed using a Personal Compound Database Library (PCDL) (Agilent, USA) with the METLIN Personal Metabolite Database and a customized database, compiled using the PCDL platform populated with compounds obtained from Naturally occurring Plant-based Anticancerous Compound-Activity-Target Database (NPACT) (29).

## Results

### Cytotoxicity comparison on SCC25 cells and HaCaT cells

To evaluate and compare the effect of leaf juice (LJ), brewed leaf juice (BLJ), and leaf decoction (LD) on cancer cell viability, the MTT assay was performed on human squamous cell carcinoma SCC25 cells and immortal non-cancerous human keratinocyte HaCaT cells, using increasing concentrations equivalent to 0.5–20.0 mg/mL of original leaf.

**Fig 1:**
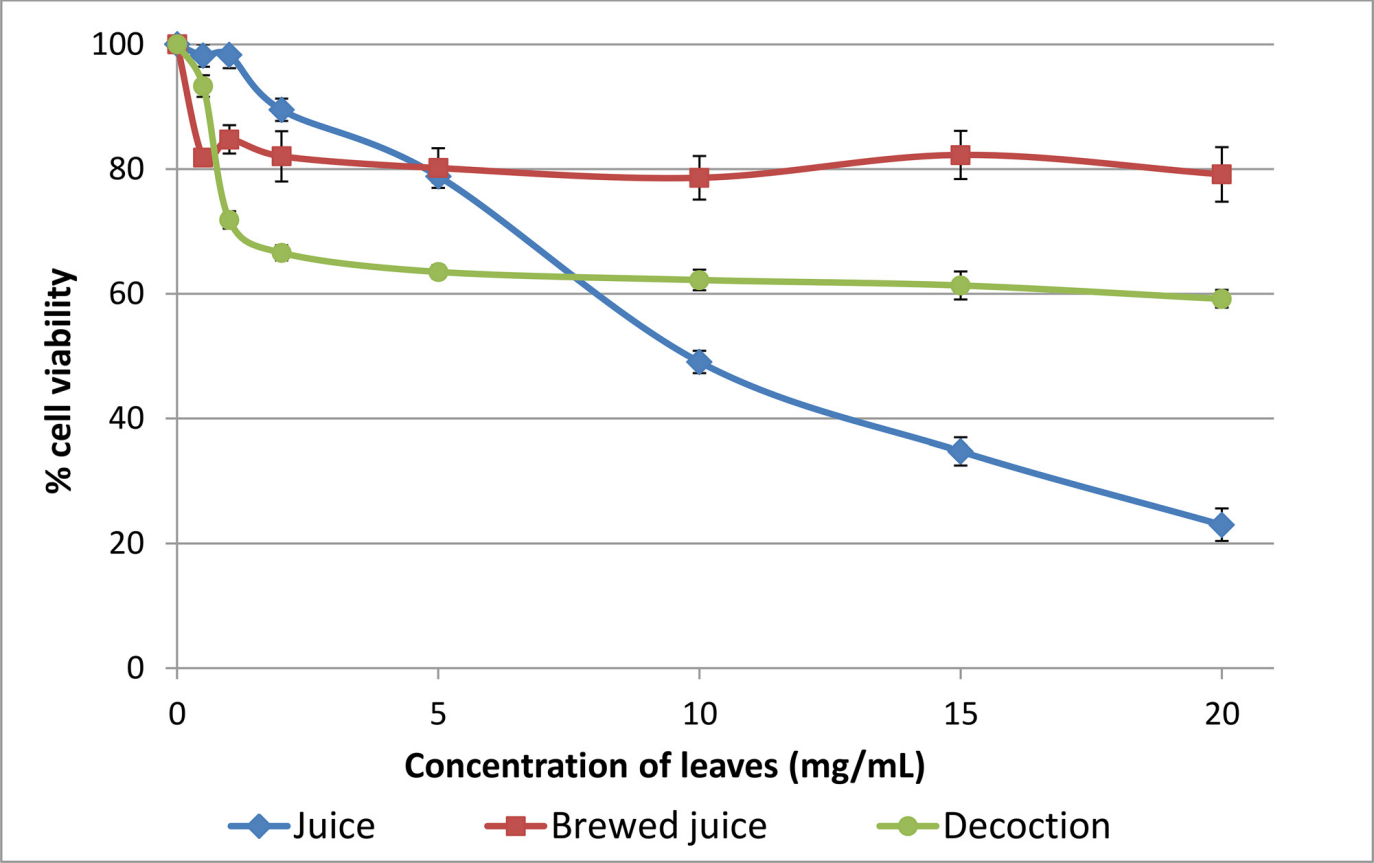
Effect of *Carica papaya* leaf extracts on the survival of SCC25 cancer cells. The cell viability was determined by the MTT assay and calculated by comparison with the control cells (exposed to medium without extracts). Results are shown as mean ± SEM (n = 3).

Fig 1 illustrates the effect of three extracts on the cell viability of SCC25 cancer cells. The brewed juice and decoction showed similar patterns of the effect, with a plateau at concentrations above 2 mg/mL, whereas the leaf juice inhibited the growth of SCC25 cells in a concentration-dependent manner (R^2^ = 0.98 for a near-linear inverse relationship between concentration and cell viability). After treatment with leaf juice at a concentration equivalent to original leaf of 20 mg/mL, only approximately 20% of SCC25 cells survived, whereas leaf decoction showed a weaker effect by reducing cell viability to a constant 60% for much of the range of tested concentrations from 2 to 20 mg/mL. Interestingly, the effect on SCC25 cells was lost when the leaf juice was brewed, with up to 80% of cells remaining viable even at the highest tested concentration.

**Fig 2:**
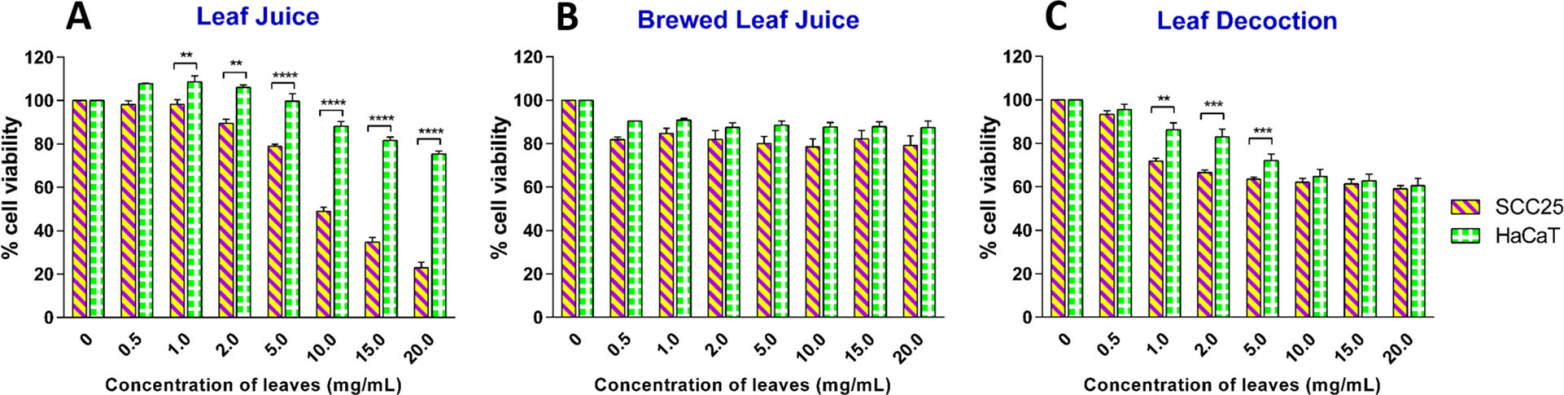
Effect of *Carica papaya* leaf extracts on the survival of SCC25 and HaCaT cells. (A) Leaf juice, (B) Brewed leaf juice, (C) Leaf decoction. Results are shown as mean ± SEM (n = 3). *, *p*<0.05; **, *p*<0.01; ***, *p*<0.001, HaCaT *vs* SCC25 (two-way ANOVA with Bonferroni post-test).

An ideal cancer preventative and therapeutic agent should specifically target the cancer cells while the non-malignant cells should not be impaired. Therefore, we selected the HaCaT cell line to perform experiments in parallel with SCC25 cells in order to examine the selective growth inhibition towards cancer cells of the extracts. When the cell viability between SCC25 cancer cells and non-cancerous HaCaT cells was compared, the leaf juice showed the most significant difference between the viability of two cell lines. At the highest concentration of test sample, approximately 20% of SCC25 cells surviving in comparison with more than 75% of non-cancerous HaCaT cells remaining viable (Fig 2). Meanwhile, the brewed leaf juice had no selective effect and the decoction only exhibited a selective effect within a low concentration range (1.0–5.0 mg/mL) whereas, at higher concentrations, the leaf decoction killed SCC25 and HaCaT cells to the same extent (~40%).

### Comparison of metabolite profiles of extracts

**Fig 3:**
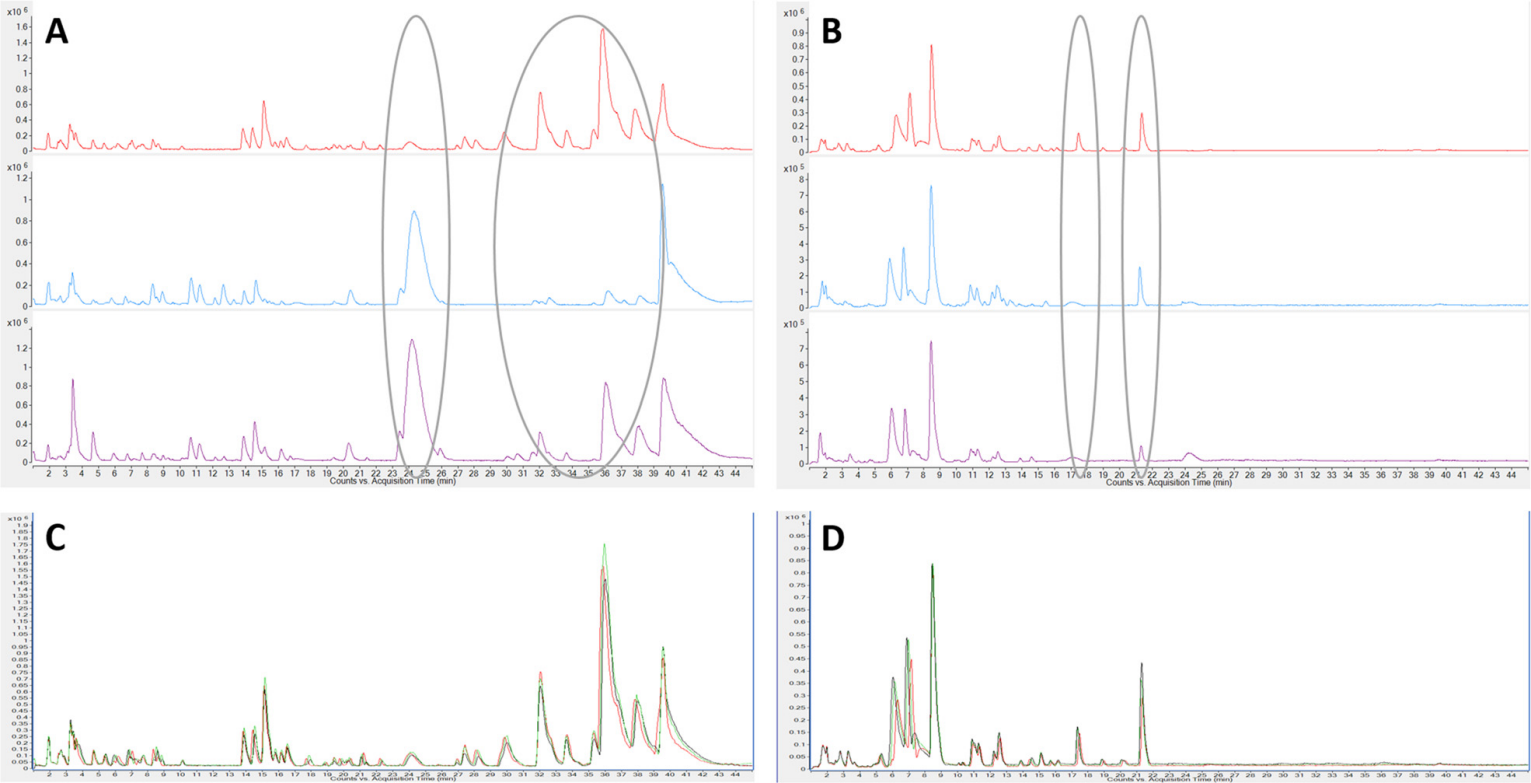
Chromatography of *Carica papaya* leaf extracts. (A) Base Peak Chromatograms (BPC) of leaf juice, brewed leaf juice, leaf decoction in positive mode and (B) in negative mode; (C) BPCs from the three replicates of leaf juice samples in positive mode and (D) in negative mode. The chromatographic region between 2 to 45 min is shown as it contained almost all of the chromatographic peaks.

The correlation between the changes in the anticancer activity of *Carica papaya* leaf juice (after being brewed) with potential changes in chemical profile initiated further exploration of the differences in the chemical profile of the three extracts. The aim was to identify the compounds that contribute to the distinct selective anticancer activity of leaf juice. Fig 3A and 3B show typical chromatograms from the LC-MS-based analysis of leaf juice, brewed leaf juice and leaf decoction in positive and negative mode, respectively. By visual comparison of base peak chromatograms in the mass range of 100 to 1700 m/z using a retention time window of 2 to 48 minutes, several differences in the profiles were detected between three extracts. In the range from 32 to 38 minutes in positive mode, LJ shows more peaks or larger peaks than BLJ and LD. In contrast, there are peaks at 24 min with higher intensities in BLJ and LD than in LJ. Similarly, the differences among the extracts are clearly observed for the peaks at about 17.5 min and 21.5 min in negative mode. Fig 3C and 3D present the consistency in the metabolic profiles of three replicates from the same leaf juice extract, highlighting the reproducibility of the acquisition.

**Fig 4:**
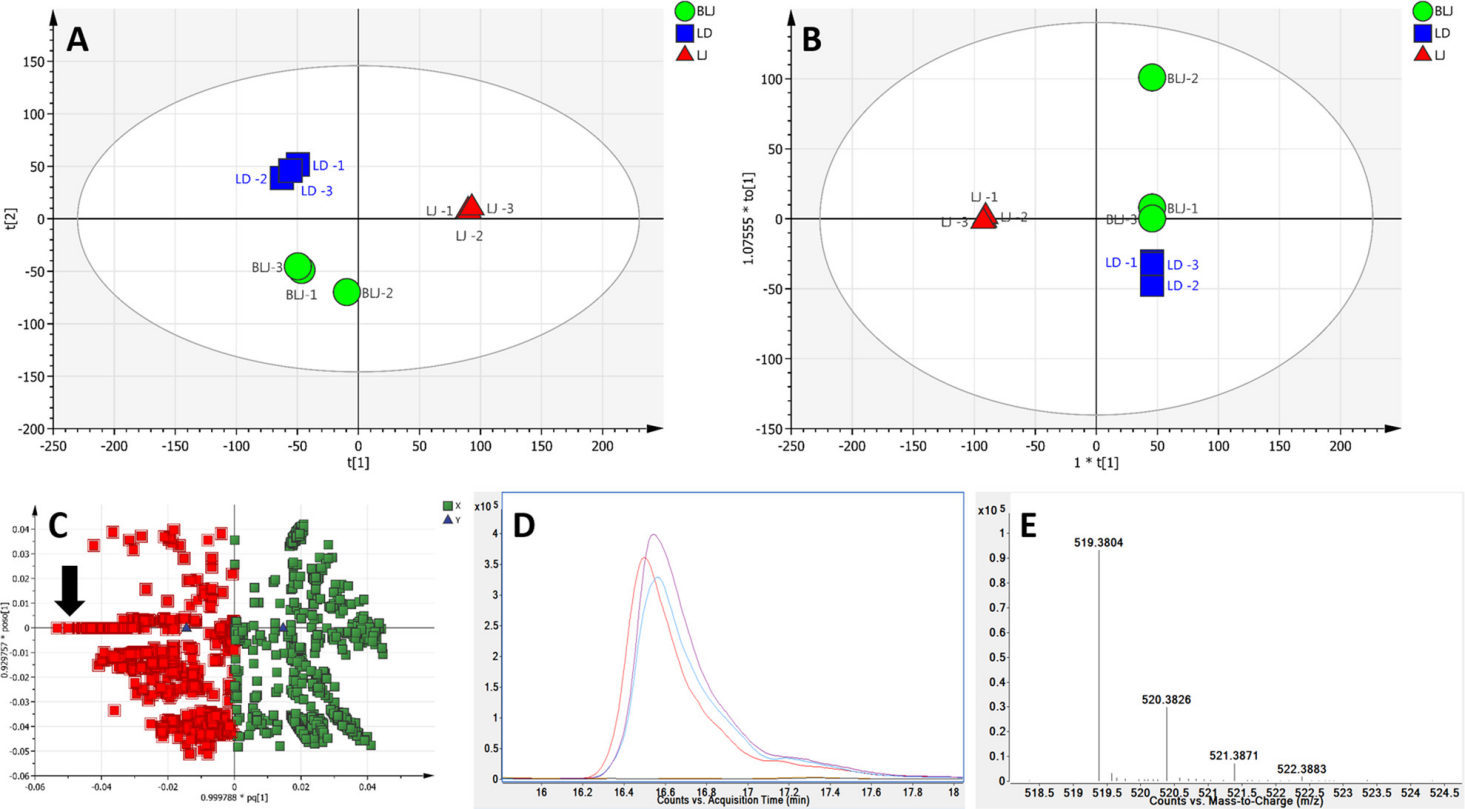
Multivariate analysis of positive ionisation LC-MS data from *Carica papaya* leaf extracts. (A) PCA scores plot, PC1 [t1] versus PC2 [t2] generated from three extracts–Leaf juice (LJ, red triangle), leaf decoction (LD, blue square) and brewed leaf juice (BLJ, green circle). Each symbol represents one replicate. (B) OPLS-DA scores plot of predictive component t[1] versus orthogonal component t_0_[1]. The ellipse in (A) and (B) represents the Hotelling’s T^2^ 95% confidence interval for the multivariate data. (C) Loadings plot derived from (B); the variables with higher abundance in LJ than BLJ/LD are highlighted in red with an arrow indicating mass 518.374@16.559 minutes. (D) Extracted ion chromatogram (EIC) of m/z 518.374 at retention time 16.559 mins. (E) Mass spectrum for EIC peak.

Unsupervised analysis by PCA was initially performed with the entire processed dataset of 1448 detected features obtained from 9 samples of 3 extracts. The multidimensional liquid-chromatography-mass spectral data were reduced to three principal components, accounting for 83.9% of the total variance. The first two component scores of the model are shown in Fig 4A. The variation between LJ samples and BLJ/LD samples are clearly defined by the first principal component (PC1). The LD and BLJ samples were also separated along the second principal component (PC2), suggesting a difference in chemical profile between samples brewed as whole leaves or as juice. However, with the main focus on the distinct biological activity of leaf juice, LD/BLJ were combined in the same group and a supervised multivariate data analysis by OPLS-DA was applied to show a more clearly defined separation between two groups: LJ and BLJ/LD (Fig 4B). The predictive component explains 50.4% of the variance in the data. The OPLS-DA model facilitated the retrieval of variables (features) responsible for the differences between two groups of extracts, as determined from the loadings plot (Fig 4C). In this plot, the variables with negative pq[1] values correspond to the metabolites/extract constituents with higher abundance in LJ, whereas the comparatively more abundant metabolites in BLJ/LD are variables with positive pq[1] values. With the aim of tentatively identifying compounds contributing to the anticancer activity of LJ, all the variables with negative pq[1] (highlighted in red) were examined by visual inspection of extracted ion chromatograms (EIC) to search for the important/discriminatory features. For example, EICs of variables with m/z 518.374 at retention time 16.559 min showed clear differences in the abundance between LJ and BLJ/LD (Fig 4D and 4E). Following interrogation of the OPLS-DA loadings plot and subsequent visual inspection and curation of the EICs, 90 metabolites in positive mode were listed as clear discriminatory features in S1 Table.

**Fig 5:**
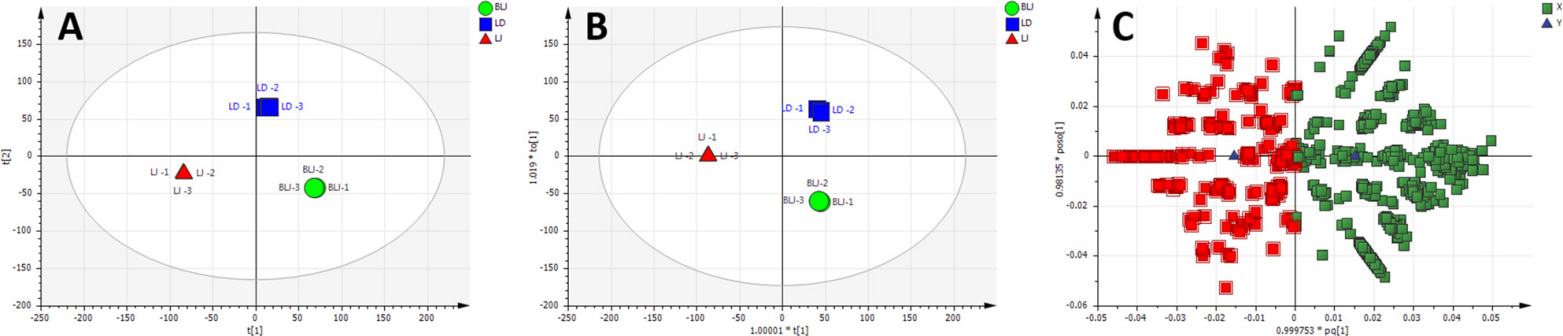
Multivariate analysis of negative ionisation LC-MS data from *Carica papaya* leaf extracts. (A) PCA scores plot, PC1 [t1] versus PC2 [t2] generated from three extracts–Leaf juice (LJ, red triangle), leaf decoction (LD, blue square) and brewed leaf juice (BLJ, green circle). Each symbol represents one replicate. (B) OPLS-DA scores plot of predictive component t[1] versus orthogonal component t_0_[1]. The ellipse in (A) and (B) represents the Hotelling’s T^2^ 95% confidence interval for the multivariate data. (C) Loadings plot derived from (B); the variables with higher abundance in LJ than BLJ/LD are highlighted in red.

Similarly, unsupervised analysis by PCA (Fig 5A) and supervised analysis by OPLS-DA (Fig 5B) were applied to the dataset of 1,500 detected features acquired in negative mode. After analysis of the loadings plot (Fig 5C) and EICs of the highlighted features with higher abundance in LJ, 104 metabolites in negative mode were listed as clear discriminatory features in S2 Table.

The accurate mass values of selected metabolites were used to generate putative empirical formulae and were then compared against METLIN Personal Metabolite Database (64,092 compounds) and the customised NPACT database (1,574 entries of plant-derived natural compounds with anticancer activity) using a mass tolerance window of ≤ 10 ppm. A total of 1,755 hits were obtained *via* the database search, for 194 features in positive and negative modes. Several features had a large number of putative compounds, for example 78 different compounds were retrieved as matches for the m/z 279.2326 in positive mode and similarly 99 different compounds corresponded to m/z 354.2405 in negative mode (S1 and S2 Tables).

### Identification of compounds by authentic standard comparison

The next step after obtaining the list of putative identifications through database searching and matching was confirmation of compound identity. In this study, we have reported the results of the direct comparison of chromatographic and spectral data to available reference compounds to confirm the exact match. Among the compounds with reported anticancer activities that were tentatively identified in papaya leaf juice, there were eleven standard compounds available in our laboratory. However, 10 out of 11 features, which were identified on the basis of accurate mass through database search, did not match to retention time of respective reference compounds as shown in Table 1 and S1 Fig.

**Table 1:** Authentic standard comparison of putative compounds in leaf juice with available reference compounds.

Test standard	Mass of Standard	Retention time of Standard (mins)	Mass of Feature	Retention time of Feature (mins)	Molecular formula	Error (ppm)
Anacardic acid	348.2664	17.20	348.2668–348.2676	19.30 & 20.64 & 25.15 & 25.83 & 31.49	C_22_H_36_O_3_	1–3
Cinnamic acid	148.0524	4.98	148.0521	3.31	C_9_H_8_O_2_	2
Dihydrocoumarin	148.0524	4.20	148.0521	3.31	C_9_H_8_O_2_	2
Dihydromethysticin	276.0998	26.12	276.0996	3.86	C_15_H_16_O_5_	1
Fucoxanthin	658.4233	40.05	658.4232	24.57	C_42_H_58_O_6_	1
Hyperforin	536.3866	37.98	536.3835	15.65	C_35_H_52_O_4_	5
Larixyl acetate	348.2664	*ND	348.2668–348.2676	19.30 & 20.64 & 25.15 & 25.83 & 31.49	C_22_H_36_O_3_	1–3
**Pheophorbide A**	**592.2686**	**33.26**	**592.2686**	**28.52 & 33.26**	**C**_**35**_**H**_**36**_**N**_**4**_**O**_**5**_	**0**
Pinolenic acid	278.2246	27.72	278.2253	21.76	C_18_H_30_O_2_	2
Stearidonic acid	276.2089	25.65	276.21	20.29	C_18_H_28_O_2_	3
Traumatic acid	228.1362	3.42	228.1362	5.38 & 5.96	C_12_H_20_O_4_	0

*ND: Not detected.

**Fig 6:**
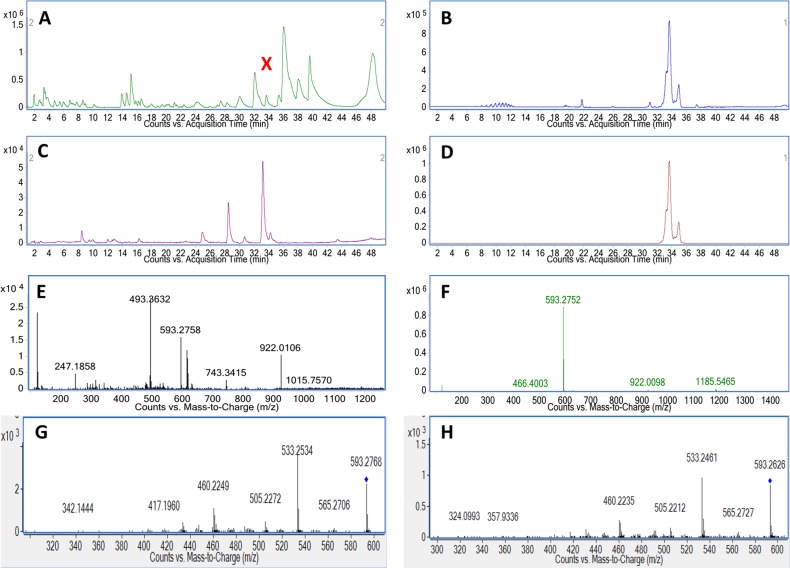
Comparison of feature 592.2686@33.257 in leaf juice against authentic standard pheophorbide A. (A) BPC chromatogram of leaf juice. (B) BPC chromatogram of pheophorbide A standard. (C) EIC of m/z 593.2752 for leaf juice. (D) EIC of m/z 593.2752 for pheophorbide A standard. (E) Mass spectrum of peak X in leaf juice. (F) Mass spectrum of pheophorbide A standard peak. (G) MS/MS spectrum of peak X in leaf juice. (H) MS/MS spectrum of pheophorbide A standard peak.

The feature 592.2686@33.257 was the only feature to be confirmed as a match with its proposed authentic standard, in this case pheophorbide A, using three properties: retention time, mass spectra and fragmentation spectra. The monoisotopic mass of the compound in leaf juice was 593.2758 [M+H]^+^ and that of pheophorbide A standard was 593.2752 [M+H]^+^ (1 ppm difference) when the retention times were matched with 33.257 min for the compound in LJ and 33.262 min for standard (Fig 6A–6F). In MS/MS mode, both peaks of the compound in leaf juice and the pheophorbide A standard produced four major fragment ions with matching m/z values: (m/z 565.2727, 533.2461, 505.2212 and 460.2235 for compound in LJ) and (m/z 565.2706, 533.2534, 505.2272 and 460.2249 for standard) (Fig 6G and 6H). The fragmentation pattern of the pheophorbide A molecule to produce those major fragments (m/z 565, 533, 505 and 460) in positive ionization mode was proposed in S2 Fig.

**Fig 7:**
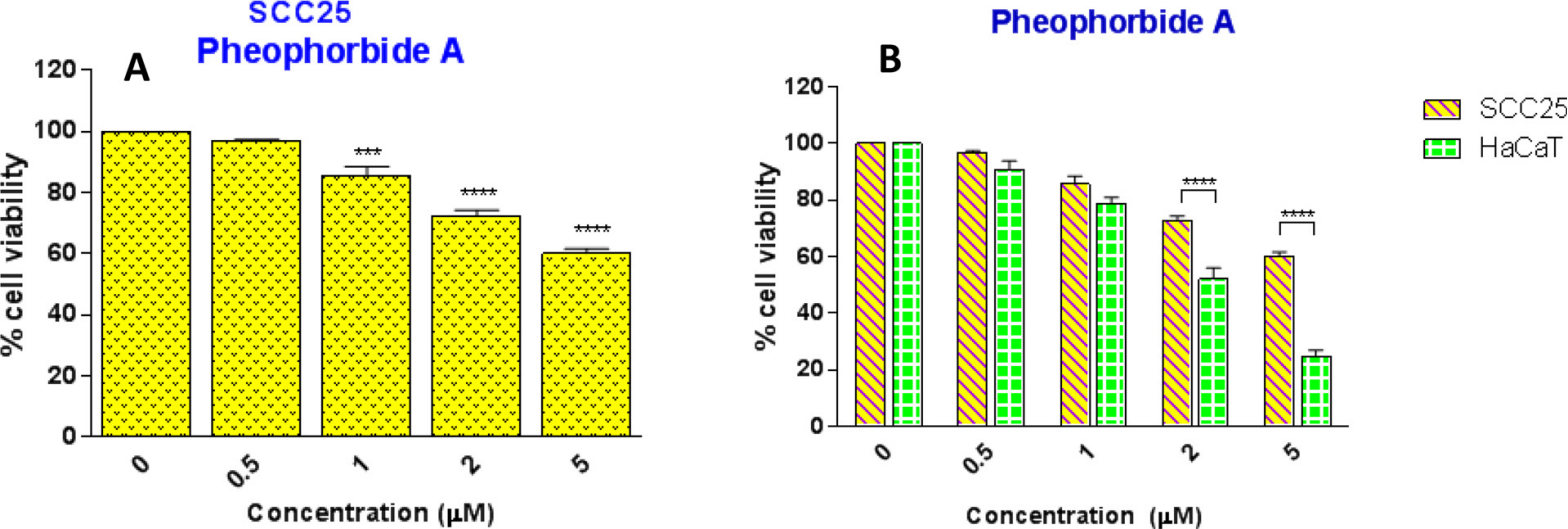
(A) Effect of pheophorbide A on the survival of SCC25 cancer cells *, *p*<0.05; **, *p*<0.01; ***, *p*<0.001, *vs* control cells (one-way ANOVA with Dunnett’s test). (B) Effect of pheophorbide A on the survival of SCC25 and HaCaT cells. *, *p*<0.05; **, *p*<0.01; ***, *p*<0.001, HaCaT *vs* SCC25 (two-way ANOVA with Bonferroni post-test). Results are shown as mean ± SEM (n = 3).

To explore the contribution of pheophorbide A to the anticancer activity of *Carica papaya* leaf juice, the MTT assay was employed to test the effect of pheophorbide A on SCC25 cancer cells and HaCaT cells. Fig 7A showed the cytotoxic activity of pheophorbide A on SCC25 cells at the range of tested concentration from 0.5 to 5 μM (the range covered the concentration of pheophorbide A found in leaf juice). However, the selective effect was not observed, as pheophorbide A exhibited higher cytotoxicity on non-cancerous HaCaT cells (Fig 7B).

## Discussion

In traditional medicine, herbal remedies are prepared in many different ways including short infusions (*e*.*g*. hot tea), longer decoctions (*e*.*g*. boiled/brewed tea), tinctures (alcohol and water extracts) and macerations (cold-soaking extracts). The processing of medicinal plants has been claimed to play several important roles in their therapeutic application: reducing toxicity and side effects, improving biological effects, changing properties or functions, preserving the active ingredients, correcting an unpleasant taste, as well as facilitating administration. In the case of *Carica papaya*, due to bitterness especially when leaves are mature, the leaves are boiled before being consumed as food or as traditional medicine. Furthermore, there are other reports relating to the side effects of fresh *Carica papaya* leaves, such as stomach irritation, purgative effects and abortion [29]. These may well be reasons why leaf decoction has been used as a traditional Aboriginal recipe for cancer treatment.

However, more recently, people in Sri Lanka, Malaysia and Pakistan have used *Carica papaya* leaf juice widely for the treatment of dengue fever and malaria with promising results [2–4]. Together with validation of pharmacological activities by confirming the significant increase of platelet count in patients with dengue fever and dengue hemorrhagic fever, several toxicity studies have been conducted to reveal that papaya leaf juice is safe for oral consumption. An acute administration study with a single dose of papaya leaf juice showed that a dose of up to 2 g/kg body weight did not cause any death or acute adverse effect on the clinical observations of the treated rats [25]. A subsequent repeat dose 28-day oral toxicity (subacute toxicity) study in rats [24] and a repeat dose 90-day oral toxicity (sub-chronic toxicity) study [26] indicated that juice with a dose equivalent of up to fourteen times the level used in traditional medicinal practice did not elicit any toxic effects.

Although our study utilised skin cancer cells, which did not require the ingestion of papaya leaves in order to elicit its effects, this information prompted us to test *Carica papaya* leaf juice for anticancer activity in order to assess this extract prior to any deleterious effects encountered during a long heating process to prepare the decoction. To the best of our knowledge, this is the first study to explore the anticancer effect of *Carica papaya* leaf juice. The effects of *Carica papaya* leaf decoction prepared by Aboriginal remedy have previously been reported being tested on the growth of different cancer cell lines: breast, stomach, lung, pancreatic, colon, liver, ovarian, cervical, neuroblastoma, lymphoma, leukaemia and other blood cancers [9, 10]. In our study, in comparison with the decoction, the leaf juice not only exhibited a stronger cytotoxic effect on SCC25 cancer cells, but also produced a clear significant selective effect when tested on non-cancerous HaCaT cells in parallel with cancer cells. Furthermore, the results demonstrating little effect of brewed leaf juice on the cancer cells and no clear selective effect between the two cell lines suggested that the brewing process reduced the selective cancer effect of *Carica papaya* leaf on squamous cell carcinoma cells SCC25 significantly. This may result from heat inactivation of temperature-sensitive bioactive compounds in the leaf juice. In general, many common metabolites of the plants such as phenolics are easily hydrolysed and oxidised under the high extraction temperature; for example anthocyanin degrades rapidly at temperature >70°C and therefore conventional extraction of anthocyanins is typically conducted at temperatures from 20 to 50°C [30]. However, there are conflicting actions of temperature in the extraction of bioactive compounds from plant materials. An increase in the extraction temperature can increase both solubility and mass transfer rate of compounds thus promoting their solubility in the solvents. In addition, at higher temperature, the extraction solvents are able to reach the sample matrices more easily due to the decrease in the viscosity and the surface tension thereby improving the extraction rate [31]. This can help to explain some weak effect exhibited by the leaf decoction, where the high temperature of brewing process for an extended period increased the solubilisation and released some other active constituents from whole leaves. With the promising results of stronger and more selective anticancer effects when compared to the Aboriginal remedy, and the above safety data, leaf juice warrants further studies to explore the activities on other cancer cell lines.

In the case of steamed ginger, an increased anti-proliferative effect was observed after steaming and subsequent exploration of the change in chemical profile allowed the elucidation of 6-shogaol as the compound of interest. 6-shogaol exhibited a remarkable increase in concentration in the samples after being steamed and was identified to be the component contributing to the improved anticancer potential [11]. We therefore applied an untargeted metabolomic approach to compare the chemical profiles of leaf juice (exhibiting selective anticancer activity) with those of leaf decoction and brewed leaf juice. Multivariate data analyses for profiles acquired by Ultra High Performance Liquid Chromatography-Quadrupole Time of Flight-Mass Spectrometry resulted in the selection of 90 and 104 features in positive and negative mode respectively.

Following feature selection, putative compound identification was obtained *via* database searching/matching. Confirmation of this “identification” presents a significant challenge. The acquisition and detection of accurate mass data permits the generation of chemical formulae but for each mass a number of structures are feasible and this number grows considerably with increased molecular weight. In particular, structural isomers cannot be differentiated by mass spectral techniques alone. For example, corresponding with the mass 278.2 and the chemical formula C_18_H_30_O_2_, there exist at least five isomers including pinolenic acid, linolenic acid, calendic acid, punicic acid and eleostearic acid; all of them have been reported to possess anticancer activities [32]. In this study, a number of features with database-derived “identification” were subjected to putative comparison with reference compounds available in our laboratory. Tellingly, only one compound (pheophorbide A) out of eleven tested standards was successfully confirmed. In light of similar research efforts and the reporting of tables of compound identities, this is of some concern [33–35]. Database searching and matching based on accurate mass data alone will provide numerous compound identities for each mass. It is therefore puzzling that such tables contain single “identities” for each mass with no supporting analytical confirmation, or reference to the multiplicity of possible additional identities.

It is thus feasible to discover several sample constituents and their associated IDs/compounds that are unique to one preparation of interest but which are not relevant to the desired activity. Conversely, many constituents and their associated/putative IDs are unique and possibly relevant but this relevance cannot be confirmed or determined without definitive identification and subsequent re-testing in the biological system.

However, despite pheophorbide A exhibiting cytotoxic activity on SCC25 cancer cells, it was found to not be the compound contributing principally to the selective activity of leaf juice. Further investigation using a wider variety of isolation, purification and identification techniques (MS^n^, NMR) are required to continue to confirm the identity of compounds contributing to the selective effect of *Carica papaya* leaf juice, particularly those altered by the long heating process applied during the traditional Aboriginal remedy preparation.

## Supporting Information

S1 Fig Authentic standard comparison with available reference compounds. (PDF)

S2 Fig Proposed MSMS fragmentation pattern of pheophorbide A in positive ionization mode. (PDF)

S1 Table List of 90 discriminatory metabolites in leaf juice extracts in positive mode. (DOCX)

S2 Table List of 104 discriminatory metabolites in leaf juice extracts in negative mode. (DOCX)
