# Supplementary material for: Traditional Aboriginal Preparation Alters the Chemical Profile of Carica papaya Leaves and Impacts on Cytotoxicity towards Human Squamous Cell Carcinoma
Source: PLoS One. 2016 Feb 1;11(2):e0147956. doi: 10.1371/journal.pone.0147956 (PMC4734615; doi:10.1371/journal.pone.0147956)
Supplement: S1 Fig — (PDF) [file pone.0147956.s001.pdf]

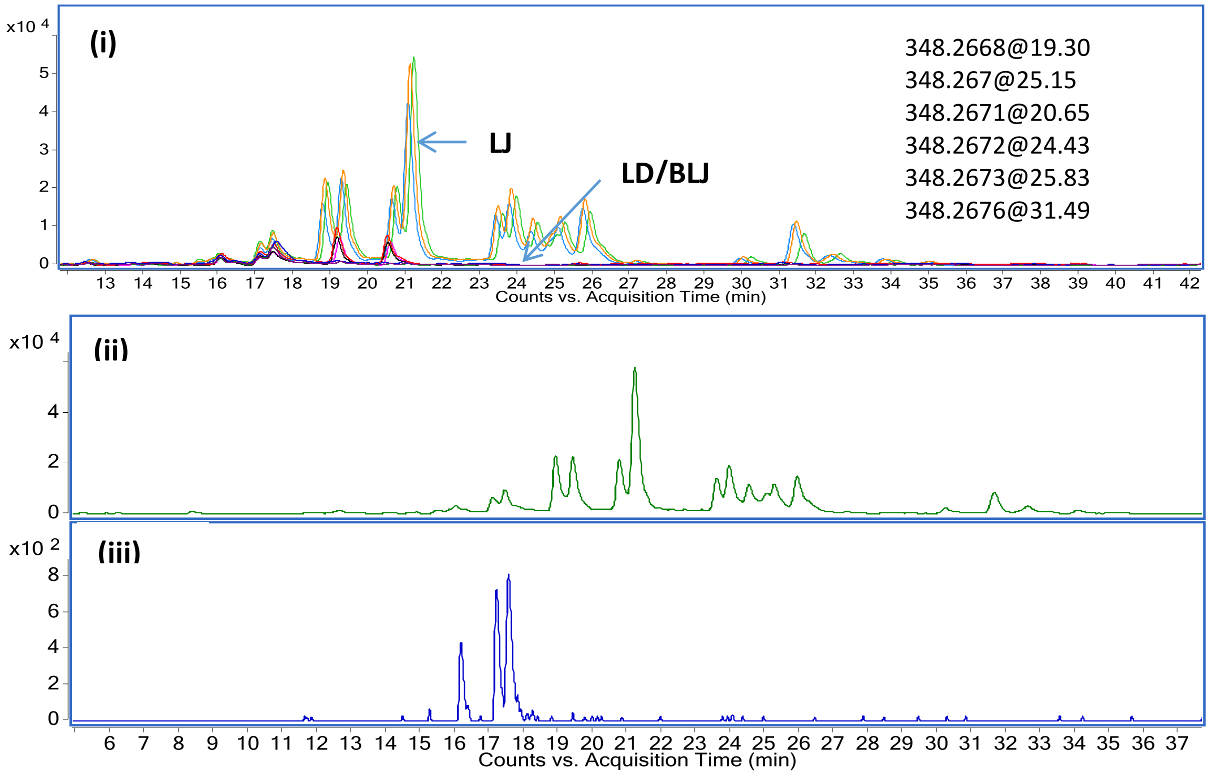


**S1A Fig. Comparison with anacardic acid standard**

(i) EIC of m/z 349.2741 for LJ, BLJ and LD extracts. (ii) EIC of m/z 349.2741 for LJ extract. (iii) EIC of m/z 349.2741 for anacardic acid standard.

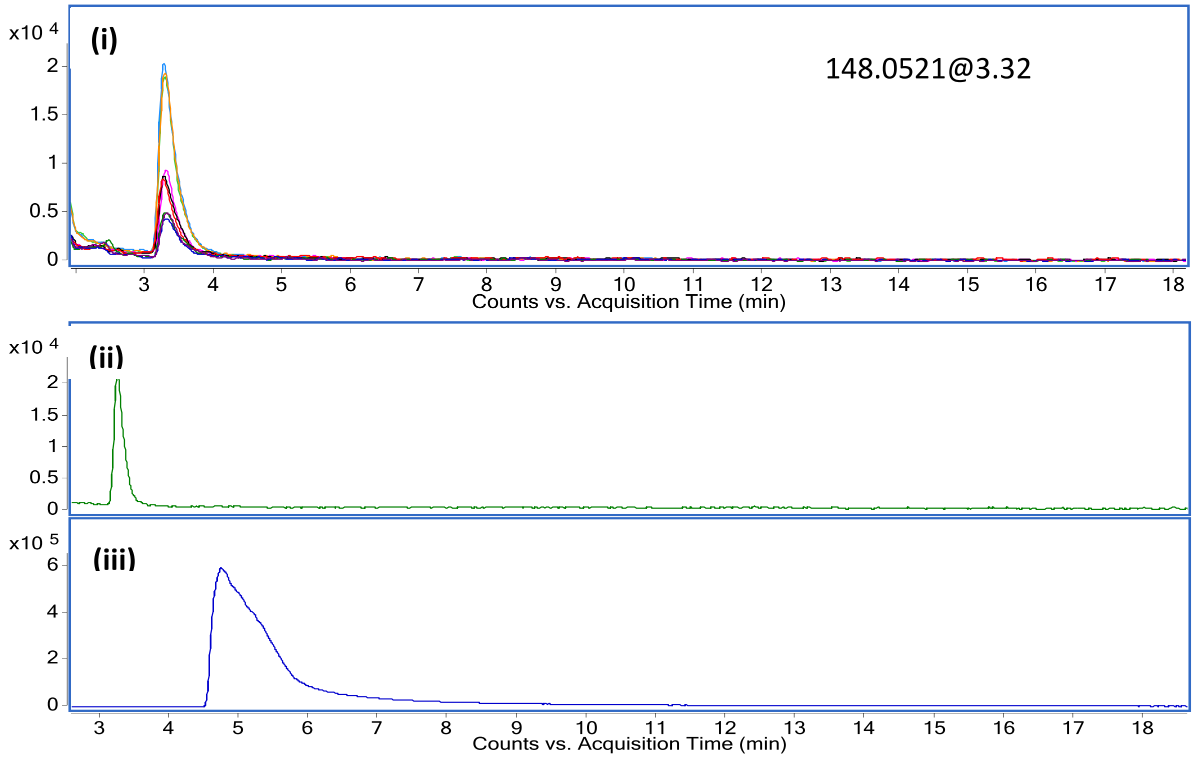


**S1B Fig. Comparison with cinnamic acid standard**

(i) EIC of m/z 147.0448 for LJ, BLJ and LD extracts. (ii) EIC of m/z 147.0448 for LJ extract. (iii) EIC of m/z 147.0448 for cinnamic acid standard.

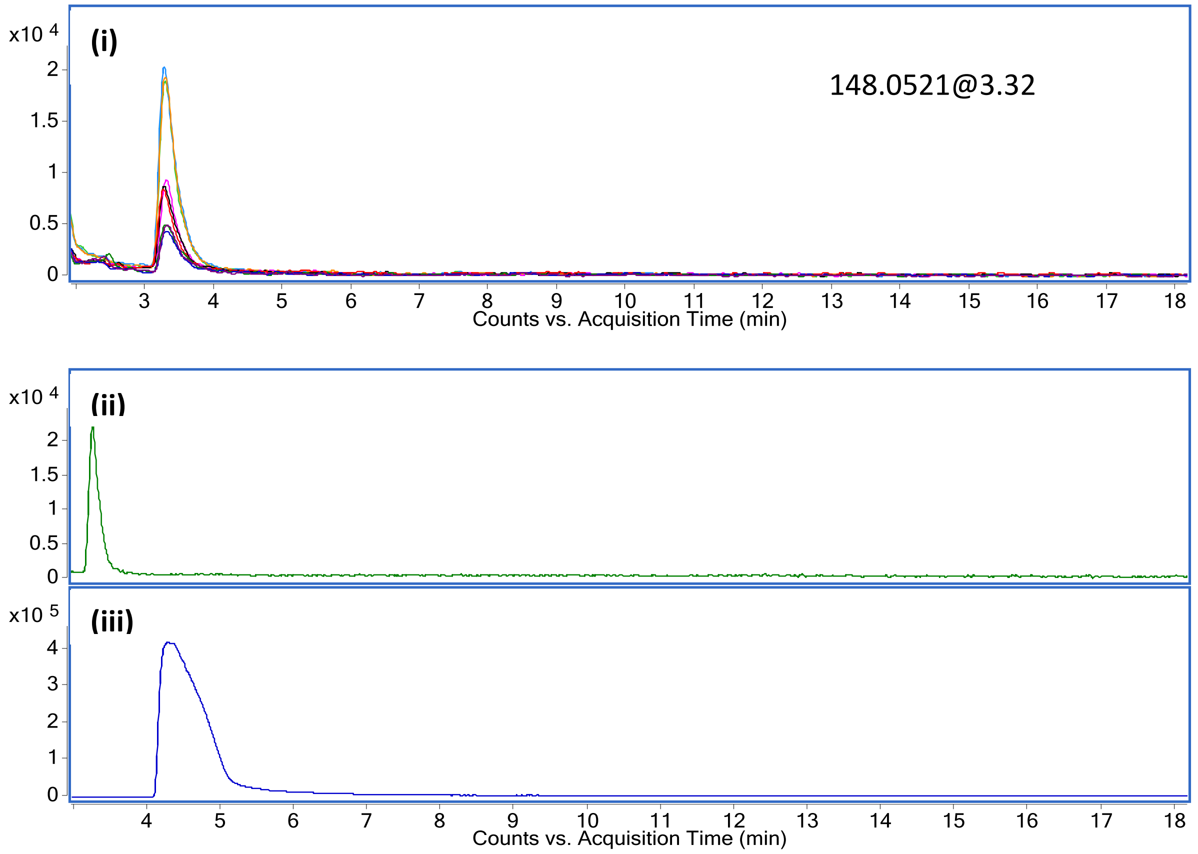


**S1C Fig. Comparison with dihydrocoumarin standard**

(i) EIC of m/z 147.0448 for LJ, BLJ and LD extracts. (ii) EIC of m/z 147.0448 for LJ extract. (iii) EIC of m/z 147.0448 for dihydrocoumarin standard

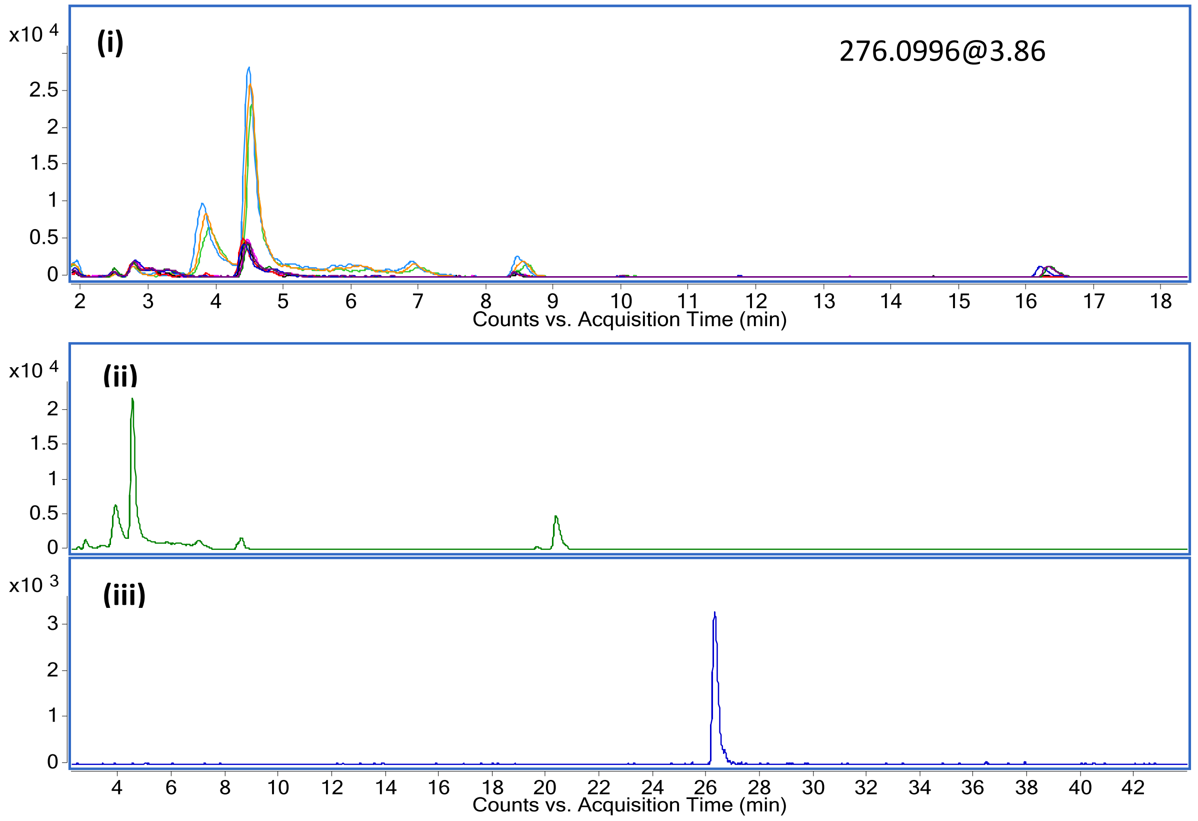


**S1D Fig. Comparison with dihydromethysticin standard**

(i) EIC of m/z 275.0923 for LJ, BLJ and LD extracts. (ii) EIC of m/z 275.0923 for LJ extract. (iii) EIC of m/z 275.0923 for dihydromethysticin standard

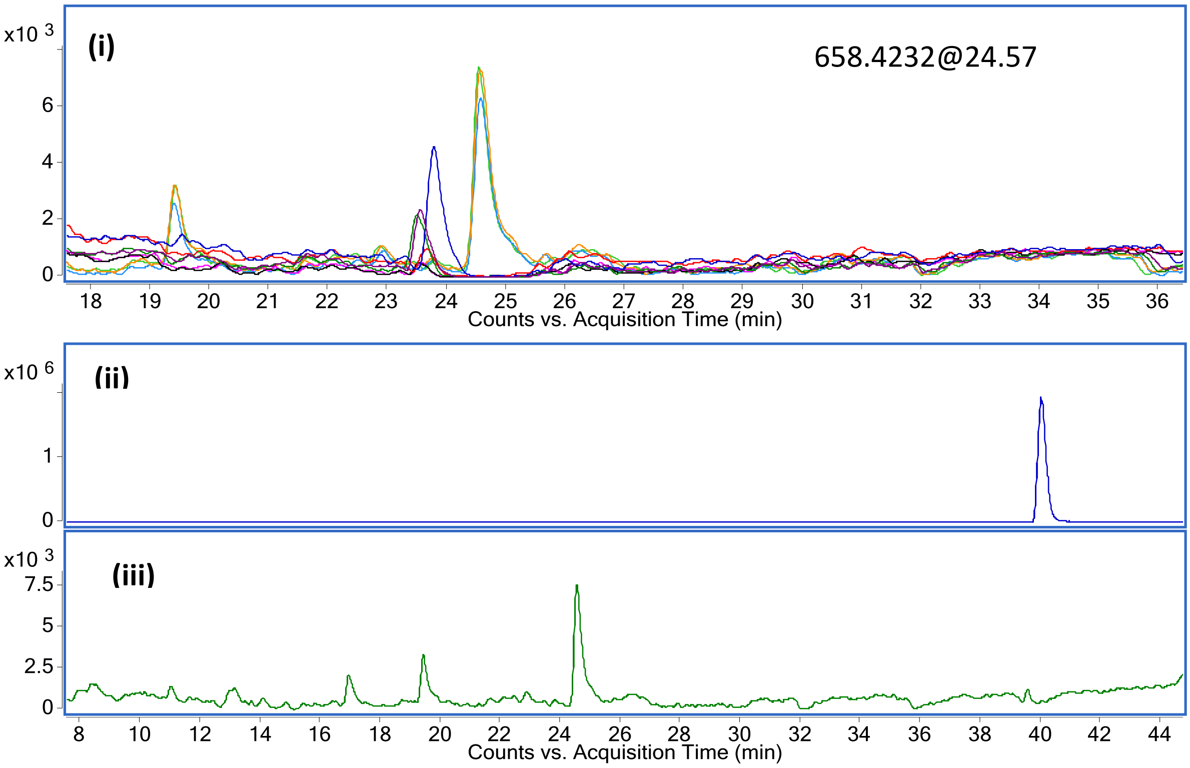


**S1E Fig. Comparison with fucoxanthin standard**

(i) EIC of m/z 659.4305 for LJ, BLJ and LD extracts. (ii) EIC of m/z 659.4305 for LJ extract. (iii) EIC of m/z 659.4305 for fucoxanthin standard

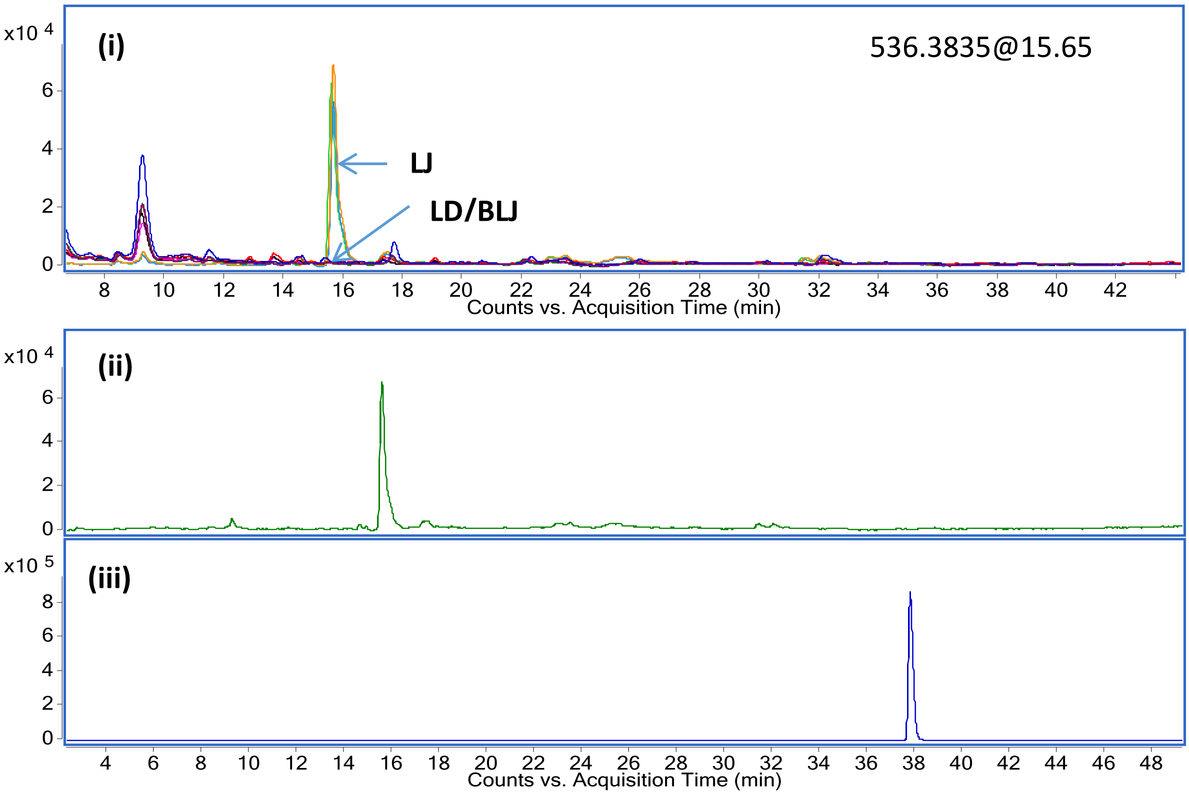


**S1F Fig. Comparison with hyperforin standard**

(i) EIC of m/z 537.3908 for LJ, BLJ and LD extracts. (ii) EIC of m/z 537.3908 for LJ extract. (iii) EIC of m/z 537.3908 for hyperforin standard.

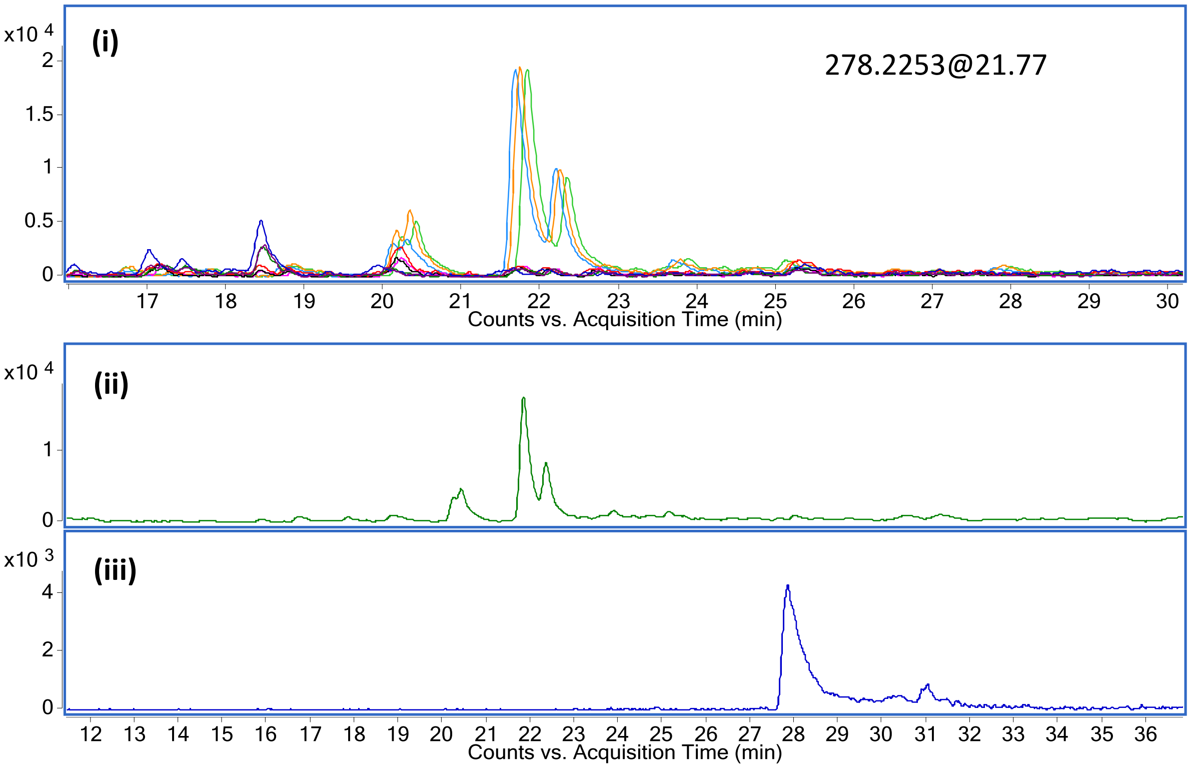


**S1G Fig. Comparison with pinolenic acid standard**

(i) EIC of m/z 279.2326 for LJ, BLJ and LD extracts. (ii) EIC of m/z 279.2326 for LJ extract. (iii) EIC of m/z 279.2326 for pinolenic acid standard.

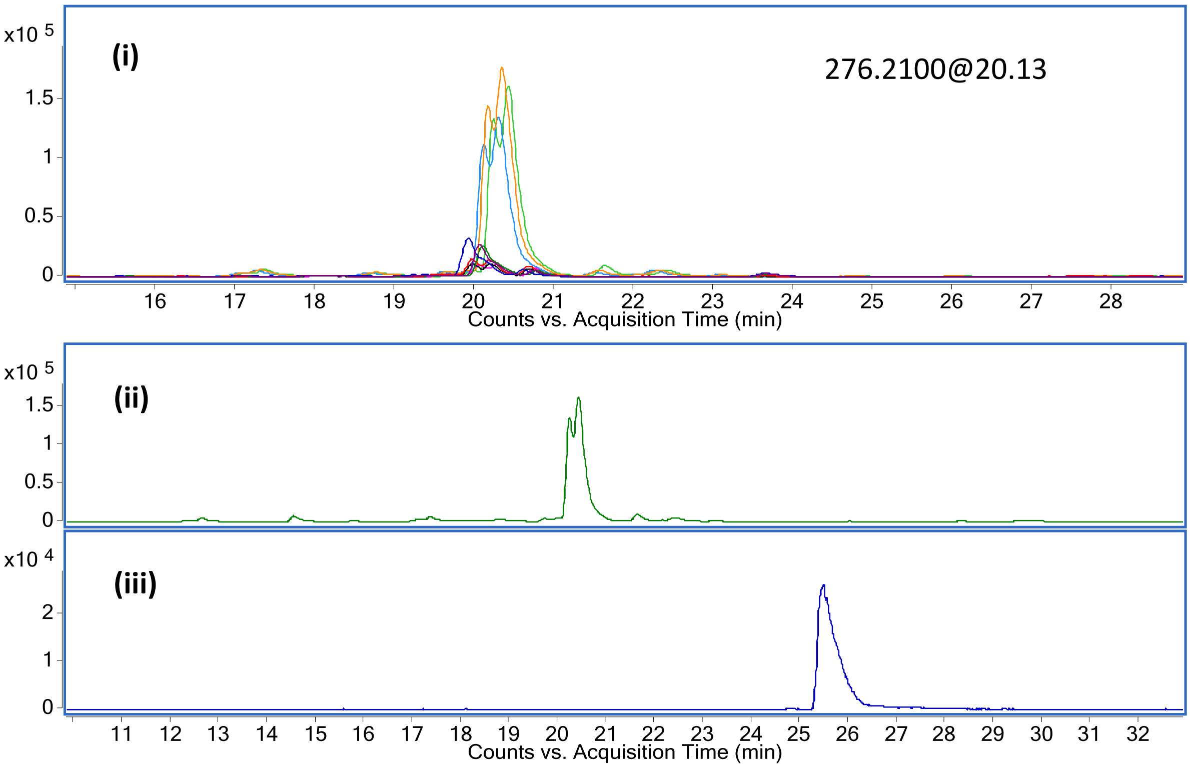


**S1H Fig. Comparison with stearidonic acid standard (positive mode)**

(i) EIC of m/z 277.2173 for LJ, BLJ and LD extracts. (ii) EIC of m/z 277.2173 for LJ extract. (iii) EIC of m/z 277.2173 for stearidonic acid standard.

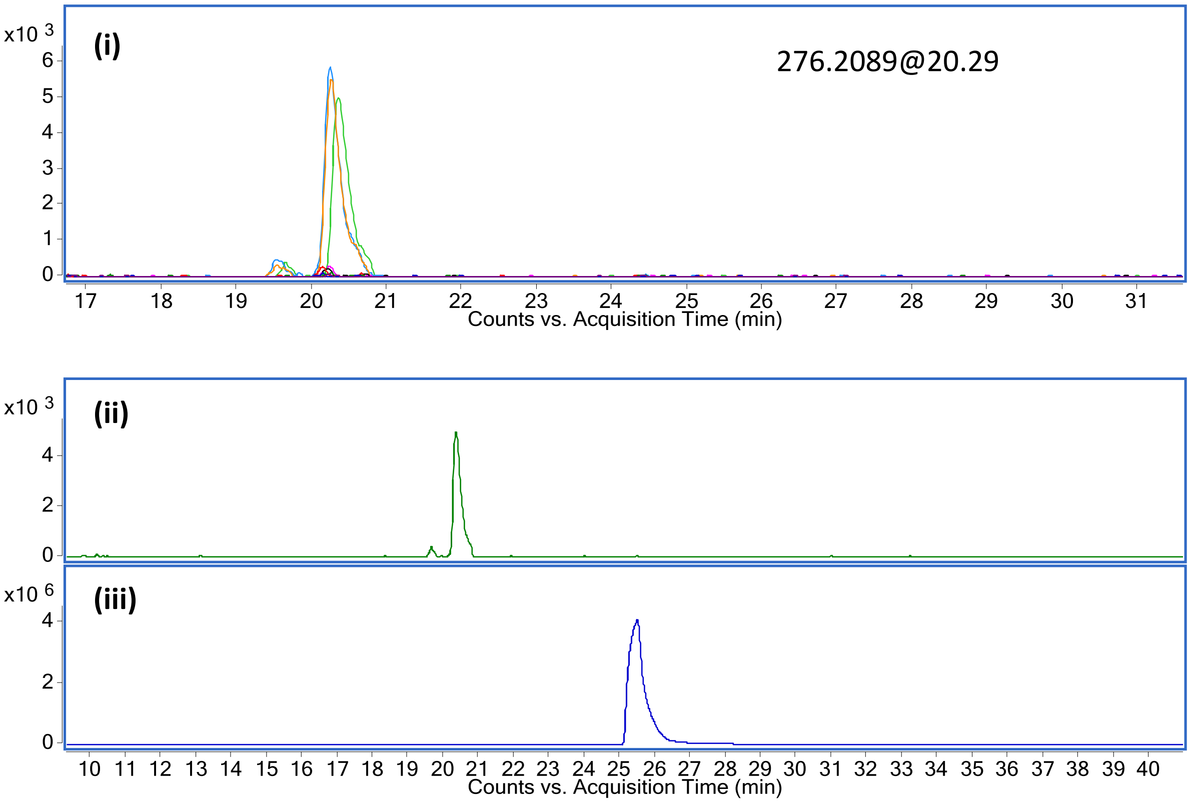


**S1K Fig. Comparison with stearidonic acid standard (negative mode)**

(i) EIC of m/z 275.2016 for LJ, BLJ and LD extracts. (ii) EIC of m/z 275.2016 for LJ extract. (iii) EIC of m/z 275.2016 for stearidonic acid standard

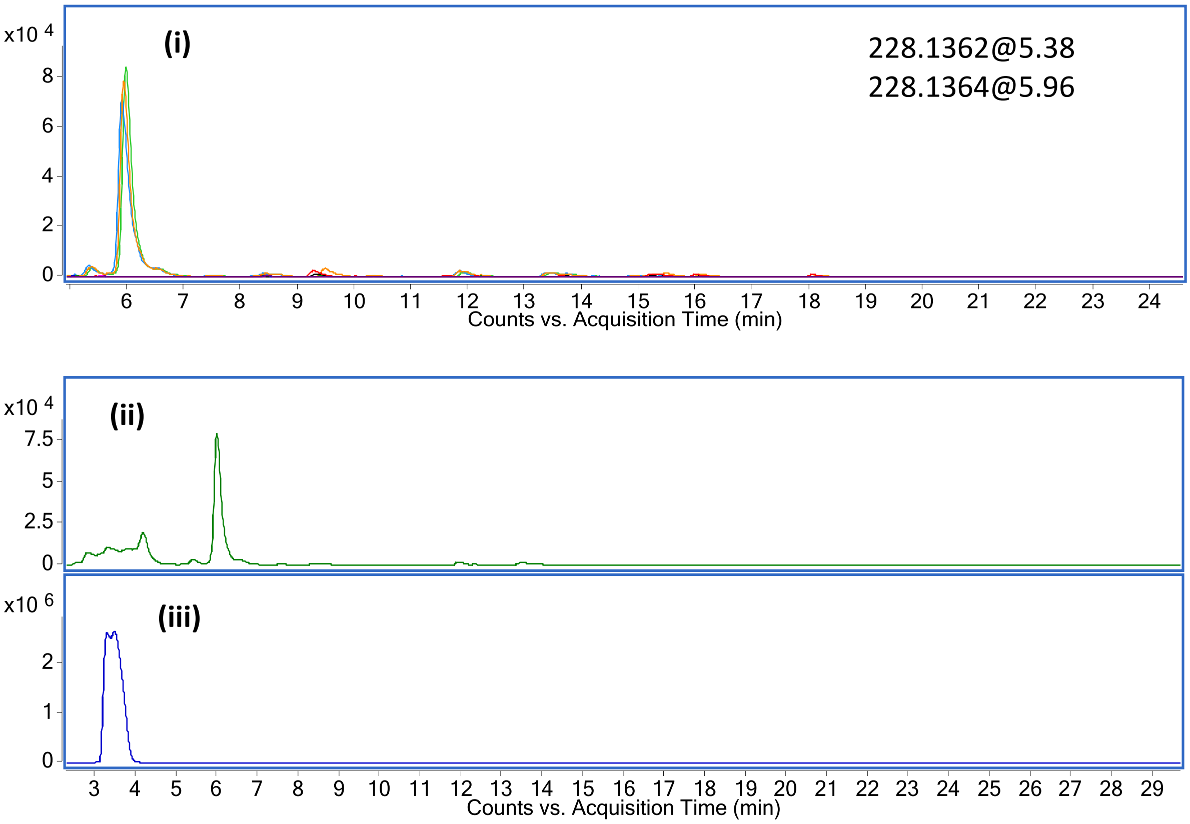


**S1L Fig. Comparison with traumatic acid standard**

(i) EIC of m/z 227.1289 for LJ, BLJ and LD extracts. (ii) EIC of m/z 227.1289 for LJ extract. (iii) EIC of m/z 227.1289 for traumatic acid standard.
