# Supplementary figures and images for: Traditional Aboriginal Preparation Alters the Chemical Profile of Carica papaya Leaves and Impacts on Cytotoxicity towards Human Squamous Cell Carcinoma
Source: PLoS One. 2016 Feb 1;11(2):e0147956. doi: 10.1371/journal.pone.0147956 (PMC4734615; doi:10.1371/journal.pone.0147956)

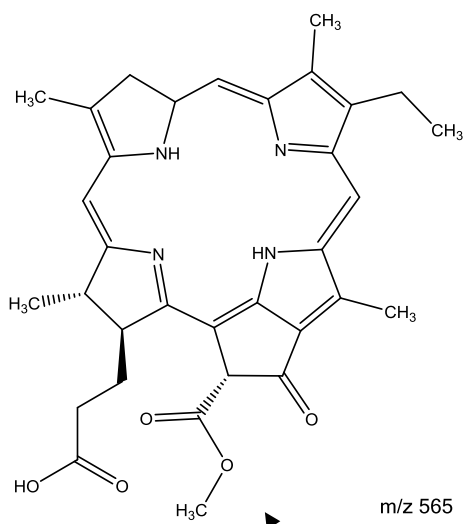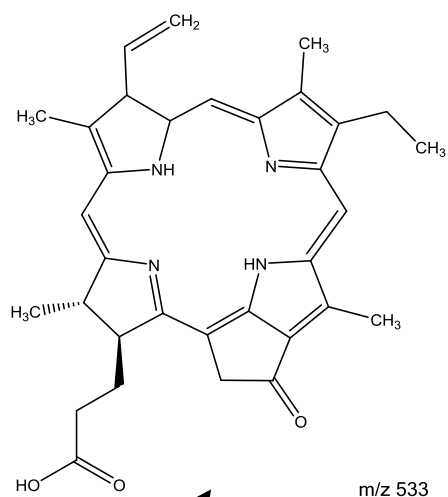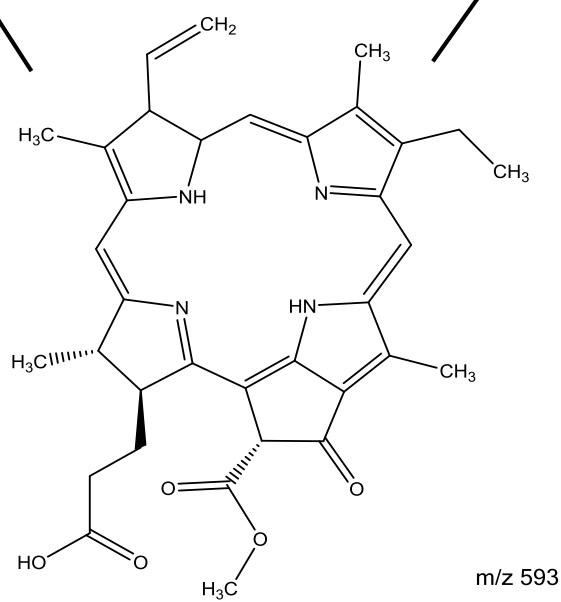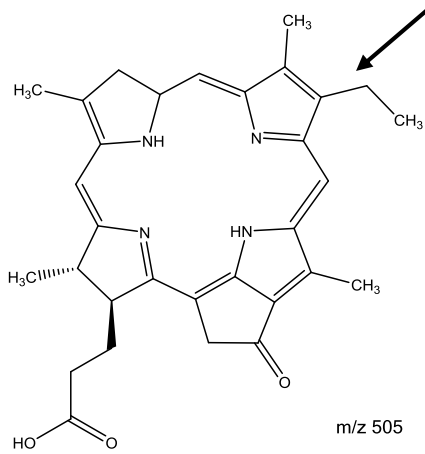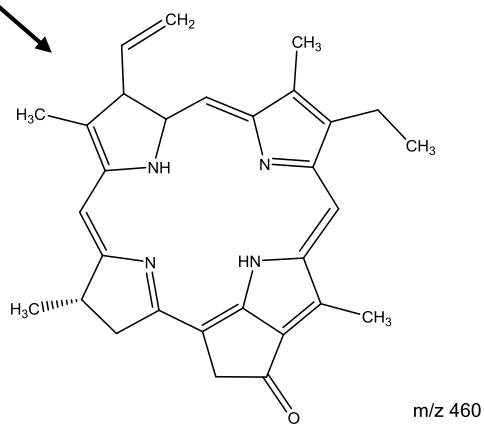

Supplement: S2 Fig — (PDF) [file pone.0147956.s002.pdf]
