# Supplementary material for: Traditional Aboriginal Preparation Alters the Chemical Profile of Carica papaya Leaves and Impacts on Cytotoxicity towards Human Squamous Cell Carcinoma
Source: PLoS One. 2016 Feb 1;11(2):e0147956. doi: 10.1371/journal.pone.0147956 (PMC4734615; doi:10.1371/journal.pone.0147956)
Supplement: S1 Table — (DOCX) [file pone.0147956.s003.docx]

| **Experimental Mass** | **Retention time** | **pq[1]** | **[M+H]^+^** | **Molecular formula** | **Error (ppm)** | **Number of hits** |
| --- | --- | --- | --- | --- | --- | --- |
| 218.1270 | 2.772 | -0.01601 | 219.1343 | C_9_H_18_N_2_O_4_ | 1 | 10 |
| 228.1474 | 4.129 | -0.00849 | 229.1547 | C_11_H_20_N_2_O_3_ | 0 | 2 |
| 230.1638 | 5.089 | -0.03722 | 231.1711 | C_11_H_23_N_2_O_3_ | 3 | 4 |
| 246.1788 | 15.822 | -0.03722 | 247.1861 | ND |  |  |
| 249.0869 | 4.864 | -0.0205 | 250.0942 | C_10_H_19_NO_2_S_2_ | 4 | 2 |
| 269.2002 | 17.858 | -0.03722 | 270.2075 | C_15_H_27_NO_3_ | 4 | 2 |
| 276.2100 | 20.128 | -0.00817 | 277.2173 | C_18_H_28_O_2_ | 3 | 74 |
| 278.2253 | 21.766 | -0.03723 | 279.2326 | C_18_H_30_O_2_ | 2 | 78 |
| 281.2001 | 5.315 | -0.03723 | 282.2074 | C_11_H_28_ClN_5_O | 6 | 1 |
| 292.2048 | 20.694 | -0.03721 | 293.2121 | C_18_H_28_O_3_ | 3 | 19 |
| 292.2048 | 15.866 | -0.03722 | 293.2121 | C_18_H_28_O_3_ | 3 | 19 |
| 292.2051 | 17.499 | -0.03723 | 293.2124 | C_18_H_28_O_3_ | 4 | 19 |
| 294.2202 | 17.244 | -0.03722 | 295.2275 | C_18_H_30_O_3_ | 2 | 44 |
| 306.2205 | 20.619 | -0.03722 | 307.2278 | C_19_H_30_O_3_ | 3 | 12 |
| 317.2006 | 22.543 | -0.03721 | 318.2079 | C_19_H_27_NO_3_ | 4 | 4 |
| 327.2419 | 15.810 | -0.03723 | 328.2492 | C_18_H_33_NO_4_ | 2 | 4 |
| 329.2572 | 17.326 | -0.03722 | 330.2645 | C_18_H_35_NO_4_ | 1 | 1 |
| 348.2668 | 19.301 | -0.0254 | 349.2741 | C_22_H_36_O_3_ | 1 | 7 |
| 348.2670 | 25.149 | -0.03722 | 349.2743 | C_22_H_36_O_3_ | 1 | 7 |
| 348.2671 | 20.646 | -0.02536 | 349.2744 | C_22_H_36_O_3_ | 1 | 7 |
| 348.2672 | 24.433 | -0.03723 | 349.2745 | C_22_H_36_O_3_ | 2 | 7 |
| 348.2673 | 25.832 | -0.03723 | 349.2746 | C_22_H_36_O_3_ | 2 | 7 |
| 348.2676 | 31.494 | -0.03722 | 349.2749 | C_22_H_36_O_3_ | 3 | 7 |
| 366.2776 | 23.495 | -0.03722 | 367.2849 | ND |  |  |
| 366.2778 | 18.859 | -0.03722 | 367.2851 | ND |  |  |
| 366.2778 | 23.858 | -0.03722 | 367.2851 | ND |  |  |
| 366.2779 | 24.957 | -0.03722 | 367.2852 | ND |  |  |
| 366.2781 | 21.147 | -0.03722 | 367.2854 | ND |  |  |
| 373.1388 | 5.509 | -0.03722 | 374.1461 | C_20_H_24_ClN_3_S | 2 | 1 |
| 381.2678 | 34.376 | -0.03722 | 382.2751 | C_18_H_40_NO_5_P | 8 | 1 |
| 394.0674 | 5.511 | -0.03723 | 395.0747 | C_21_H_14_O_8_ | 3 | 4 |
| 456.1160 | 8.384 | -0.03722 | 457.1233 | ND |  |  |
| 458.2077 | 24.602 | -0.03723 | 459.2150 | C_24_H_30_N_2_O_7_ | 5 | 1 |
| 490.3416 | 29.234 | -0.03722 | 491.3489 | C_33_H_46_O_3_ | 6 | 1 |
| 490.3419 | 26.945 | -0.0316 | 491.3492 | C_33_H_46_O_3_ | 5 | 1 |
| 490.3420 | 27.455 | -0.01512 | 491.3493 | C_33_H_46_O_3_ | 5 | 1 |
| 492.3577 | 15.195 | -0.00937 | 493.3650 | C_33_H_48_O_3_ | 5 | 2 |
| 500.3622 | 37.079 | -0.03722 | 501.3695 | C_30_H_48_N_2_O_4_ | 1 | 1 |
| 500.3630 | 35.385 | -0.03249 | 501.3703 | C_30_H_48_N_2_O_4_ | 3 | 1 |
| 502.3786 | 39.863 | -0.03722 | 503.3859 | ND |  |  |
| 503.2376 | 6.958 | -0.03723 | 504.2449 | C_28_H_40_O_8_  C_29_H_36_N_4_O_4_ | 4  6 | 5  1 |
| 504.2703 | 21.026 | -0.03722 | 505.2776 | ND |  |  |
| 508.3524 | 13.774 | -0.03723 | 509.3597 | ND |  |  |
| 516.3561 | 32.901 | -0.03722 | 517.3634 | ND |  |  |
| 516.3570 | 30.175 | -0.03722 | 517.3643 | ND |  |  |
| 518.3712 | 26.051 | -0.03723 | 519.3785 | ND |  |  |
| 518.3721 | 11.908 | -0.03723 | 519.3794 | ND |  |  |
| 518.3727 | 33.016 | -0.03723 | 519.3800 | ND |  |  |
| 518.3727 | 37.749 | -0.03723 | 519.3800 | ND |  |  |
| 518.3729 | 32.371 | -0.03723 | 519.3802 | ND |  |  |
| 518.3740 | 16.559 | -0.03723 | 519.3813 | ND |  |  |
| 520.3886 | 15.931 | -0.03722 | 521.3959 | C_35_H_52_O_3_ | 5 | 1 |
| 520.3886 | 17.721 | -0.03723 | 521.3959 | C_35_H_52_O_3_ | 5 | 1 |
| 526.3782 | 38.838 | -0.03722 | 527.3855 | ND |  |  |
| 536.3835 | 15.649 | -0.03722 | 537.3908 | C_35_H_52_O_4_ | 5 | 1 |
| 570.4043 | 30.793 | -0.03723 | 571.4116 | ND |  |  |
| 572.4199 | 33.242 | -0.03722 | 573.4272 | ND |  |  |
| 592.2686 | 33.257 | -0.03722 | 593.2759 | C_29_H_40_N_2_O_11_  C_27_H_45_O_12_P  C_35_H_36_N_4_O_5_ | 9  6  0 | 1  1  1 |
| 592.2687 | 28.522 | -0.03722 | 593.2760 | C_29_H_40_N_2_O_11_  C_27_H_45_O_12_P  C_35_H_36_N_4_O_5_ | 9  6  0 | 1  1  1 |
| 592.3726 | 24.854 | -0.03722 | 593.3799 | C_37_H_52_O_6_  C_38_H_48_N_4_O_2_ | 6  8 | 1  1 |
| 614.2349 | 28.523 | -0.03722 | 615.2422 | C_35_H_34_N_4_O_5_ | 4 | 2 |
| 630.3521 | 20.668 | -0.03722 | 631.3594 | C_39_H_50_O_7_ | 5 | 1 |
| 632.3675 | 19.871 | -0.03722 | 633.3748 | C_40_H_48_N_4_O_3_ | 8 | 1 |
| 648.3626 | 23.450 | -0.03722 | 649.3699 | ND |  |  |
| 650.2550 | 14.072 | -0.03722 | 651.2623 | C_39_H_38_O_9_  C_32_H_38_N_6_O_7_S  C_32_H_42_O_14_ | 5  1  3 | 1  1  2 |
| 650.3782 | 15.881 | -0.03722 | 651.3855 | C_39_H_54_O_8_ | 5 | 2 |
| 650.3789 | 22.323 | -0.03722 | 651.3862 | C_39_H_54_O_8_ | 5 | 2 |
| 658.4232 | 24.566 | -0.03723 | 659.4305 | C_42_H_58_O_6_ | 1 | 1 |
| 661.4609 | 20.237 | -0.03722 | 662.4682 | C_35_H_67_NO_8_S | 1 | 1 |
| 663.1115 | 1.809 | -0.03722 | 664.1188 | C_21_H_27_N_7_O_14_P_2_ | 3 | 2 |
| 665.2897 | 6.320 | -0.03723 | 666.2970 | ND |  |  |
| 684.4712 | 26.276 | -0.03722 | 685.4785 | C_38_H_69_O_8_P | 2 | 12 |
| 684.4716 | 29.359 | -0.03722 | 685.4789 | C_38_H_69_O_8_P | 2 | 12 |
| 684.4720 | 22.011 | -0.03722 | 685.4793 | C_38_H_69_O_8_P | 1 | 12 |
| 684.4721 | 20.107 | -0.03723 | 685.4794 | C_38_H_69_O_8_P | 1 | 12 |
| 686.4874 | 20.407 | -0.03722 | 687.4947 | C_38_H_71_O_8_P | 1 | 16 |
| 705.4864 | 20.379 | -0.03722 | 706.4937 | ND |  |  |
| 742.3362 | 15.253 | -0.01875 | 743.3435 | C_35_H_50_N_8_O_6_S_2_ | 9 | 1 |
| 742.3363 | 28.221 | -0.03722 | 743.3436 | C_35_H_50_N_8_O_6_S_2_ | 9 | 1 |
| 742.3365 | 29.984 | -0.03017 | 743.3438 | C_35_H_50_N_8_O_6_S_2_ | 9 | 1 |
| 749.5133 | 20.510 | -0.03722 | 750.5206 | C_39_H_76_NO_10_P | 9 | 10 |
| 750.3415 | 35.361 | -0.03723 | 751.3488 | ND |  |  |
| 768.3525 | 16.556 | -0.03723 | 769.3598 | ND |  |  |
| 793.5397 | 20.636 | -0.03722 | 794.5470 | ND |  |  |
| 814.4696 | 20.636 | -0.03722 | 815.4769 | C_42_H_71_O_13_P  C_42_H_70_O_15_ | 7  2 | 4  1 |
| 837.5664 | 20.752 | -0.03722 | 838.5737 | C_47_H_75_N_5_O_8_ | 5 | 1 |
| 858.4956 | 20.749 | -0.03722 | 859.5029 | ND |  |  |
| 881.5911 | 20.858 | -0.03723 | 882.5984 | C_52_H_84_NO_8_P | 2 | 6 |
| 946.5479 | 20.960 | -0.03722 | 947.5552 | C_48_H_82_O_18_ | 2 | 4 |
| 1283.724 | 14.106 | -0.03722 | 1284.7313 | ND |  |  |

*ND: Not determined
