# Supplementary material for: Traditional Aboriginal Preparation Alters the Chemical Profile of Carica papaya Leaves and Impacts on Cytotoxicity towards Human Squamous Cell Carcinoma
Source: PLoS One. 2016 Feb 1;11(2):e0147956. doi: 10.1371/journal.pone.0147956 (PMC4734615; doi:10.1371/journal.pone.0147956)
Supplement: S2 Table — (DOCX) [file pone.0147956.s004.docx]

| **Experimental Mass** | **Retention time** | **pq[1]** | **[M-H]^-^** | **Molecular formula** | **Error (ppm)** | **Number of hits** |
| --- | --- | --- | --- | --- | --- | --- |
| 131.0935 | 2.620 | -0.02883 | 130.0862 | C_6_H_13_NO_2_ | 8 | 15 |
| 144.1241 | 2.803 | -0.03378 | 143.1168 | ND |  |  |
| 146.0587 | 1.919 | -0.02235 | 145.0514 | C_6_H_10_O_4_ | 5 | 18 |
| 146.1048 | 2.210 | -0.03377 | 145.0975 | C_6_H_14_N_2_O_2_ | 4 | 7 |
| 147.0438 | 1.869 | -0.00772 | 146.0365 | ND |  |  |
| 148.0245 | 1.777 | -0.03373 | 147.0172 | ND |  |  |
| 148.0521 | 3.312 | -0.00719 | 147.0448 | C_9_H_8_O_2_ | 2 | 9 |
| 149.0503 | 2.450 | -0.02330 | 148.043 | C_5_H_11_NO_2_S | 5 | 3 |
| 165.0788 | 3.312 | -0.02668 | 164.0715 | C_9_H_11_NO_2_ | 1 | 15 |
| 174.0956 | 1.965 | -0.02627 | 173.0883 | ND |  |  |
| 188.1137 | 2.823 | -0.02098 | 187.1064 | ND |  |  |
| 188.1148 | 1.999 | -0.03378 | 187.1075 | C_8_H_16_N_2_O_3_ | 6 | 9 |
| 188.1158 | 2.497 | -0.03486 | 187.1085 | C_8_H_16_N_2_O_3_ | 6 | 9 |
| 204.1048 | 1.958 | -0.02549 | 203.0975 | ND |  |  |
| 214.1568 | 10.338 | -0.03377 | 213.1495 | C_12_H_22_O_3_ | 0 | 15 |
| 216.1471 | 3.324 | -0.00857 | 215.1398 | C_10_H_20_N_2_O_3_ | 1 | 1 |
| 218.1237 | 2.765 | -0.03378 | 217.1164 | ND |  |  |
| 218.1258 | 2.503 | -0.02262 | 217.1185 | C_9_H_18_N_2_O_4_ | 3 | 10 |
| 226.1204 | 7.518 | -0.03377 | 225.1131 | C_12_H_18_O_4_ | 0 | 7 |
| 228.1362 | 5.377 | -0.03377 | 227.1289 | C_12_H_20_O_4_ | 0 | 7 |
| 228.1364 | 5.961 | -0.03378 | 227.1291 | C_12_H_20_O_4_ | 1 | 7 |
| 230.1632 | 5.339 | -0.03116 | 229.1559 | C_11_H_22_N_2_O_3_ | 0 | 4 |
| 232.1396 | 2.833 | -0.00761 | 231.1323 | ND |  |  |
| 242.1255 | 3.195 | -0.02459 | 241.1182 | C_11_H_18_N_2_O_4_ | 4 | 3 |
| 244.1781 | 7.192 | -0.03377 | 243.1708 | C_12_H_24_N_2_O_3_ | 2 | 6 |
| 244.1789 | 7.683 | -0.03392 | 243.1716 | C_12_H_24_N_2_O_3_ | 0 | 6 |
| 245.172 | 2.511 | -0.03377 | 244.1647 | C_11_H_23_N_3_O_3_ | 7 | 2 |
| 248.1192 | 3.752 | -0.03029 | 247.1119 | C_10_H_20_N_2_O_3_S | 1 | 2 |
| 254.1365 | 2.508 | -0.03377 | 253.1292 | C_14_H_22_O_2_S  C_11_H_18_N_4_O_3_ | 9  5 | 2  5 |
| 259.1612 | 2.770 | -0.03378 | 258.1539 | ND |  |  |
| 259.187 | 2.908 | -0.03377 | 258.1797 | C_12_H_25_N_3_O_3_ | 9 | 4 |
| 260.1205 | 1.888 | -0.03378 | 259.1132 | ND |  |  |
| 262.1348 | 5.904 | -0.03289 | 261.1275 | C_11_H_22_N_2_O_3_S  C_19_H_18_O | 1  3 | 4  1 |
| 264.1475 | 7.581 | -0.03424 | 263.1402 | C_14_H_20_N_2_O_3_ | 0 | 5 |
| 271.1781 | 3.942 | -0.02186 | 270.1708 | ND |  |  |
| 276.0996 | 3.859 | -0.03377 | 275.0923 | C_15_H_16_O_5_ | 0 | 9 |
| 276.111 | 4.481 | -0.02385 | 275.1037 | ND |  |  |
| 276.2089 | 20.293 | -0.03377 | 275.2016 | C_18_H_28_O_2_ | 0 | 74 |
| 292.2039 | 21.197 | -0.03377 | 291.1966 | C_18_H_28_O_3_ | 0 | 19 |
| 294.1042 | 1.952 | -0.03377 | 293.0969 | C_10_H_18_N_2_O_8_ | 7 | 3 |
| 294.2195 | 23.059 | -0.03376 | 293.2122 | C_18_H_30_O_3_ | 0 | 44 |
| 294.2197 | 22.286 | -0.03378 | 293.2124 | C_18_H_30_O_3_ | 0 | 44 |
| 294.2197 | 20.056 | -0.02652 | 293.2124 | C_18_H_30_O_3_ | 0 | 44 |
| 296.2351 | 22.181 | -0.03378 | 295.2278 | C_18_H_32_O_3_ | 0 | 52 |
| 296.2354 | 21.682 | -0.03378 | 295.2281 | C_18_H_32_O_3_ | 0 | 52 |
| 308.199 | 15.116 | -0.03377 | 307.1917 | C_16_H_27_F_3_O_2_ | 8 | 2 |
| 310.2144 | 16.464 | -0.02346 | 309.2071 | C_18_H_30_O_4_ | 0 | 25 |
| 310.2145 | 18.215 | -0.03361 | 309.2072 | C_18_H_30_O_4_ | 0 | 25 |
| 310.2145 | 14.820 | -0.03002 | 309.2072 | C_18_H_30_O_4_ | 0 | 25 |
| 310.2146 | 15.708 | -0.03182 | 309.2073 | C_18_H_30_O_4_ | 0 | 25 |
| 310.2151 | 17.295 | -0.02310 | 309.2078 | C_18_H_30_O_4_ | 2 | 25 |
| 312.2297 | 17.246 | -0.03376 | 311.2224 | C_18_H_32_O_4_ | 1 | 51 |
| 312.23 | 16.175 | -0.01626 | 311.2227 | C_18_H_32_O_4_ | 0 | 51 |
| 312.23 | 16.760 | -0.01046 | 311.2227 | C_18_H_32_O_4_ | 0 | 51 |
| 312.2302 | 18.203 | -0.03378 | 311.2229 | C_18_H_32_O_4_ | 0 | 51 |
| 312.2303 | 18.916 | -0.03377 | 311.223 | C_18_H_32_O_4_ | 0 | 51 |
| 314.2455 | 18.641 | -0.03377 | 313.2382 | C_18_H_34_O_4_ | 0 | 43 |
| 314.2458 | 17.677 | -0.02581 | 313.2385 | C_18_H_34_O_4_ | 0 | 43 |
| 316.2617 | 20.420 | -0.03377 | 315.2544 | C_18_H_36_O_4_ | 18 | 43 |
| 324.2303 | 20.603 | -0.03377 | 323.223 | C_19_H_32_O_4_ | 0 | 5 |
| 326.2091 | 11.883 | -0.03377 | 325.2018 | C_18_H_30_O_5_ | 0 | 8 |
| 326.2094 | 12.288 | -0.03377 | 325.2021 | C_18_H_30_O_5_ | 0 | 8 |
| 338.2091 | 16.609 | -0.03376 | 337.2018 | C_20_H_28_F_2_O_2_  C_19_H_30_O_5_  C_20_H_26_N_4_O | 9  0  4 | 1  7  1 |
| 340.2251 | 14.966 | -0.03377 | 339.2178 | C_19_H_32_O_5_ | 0 | 2 |
| 342.2397 | 13.928 | -0.03378 | 341.2324 | C_19_H_34_O_5_ | 2 | 4 |
| 342.2403 | 16.940 | -0.03377 | 341.233 | C_19_H_34_O_5_ | 0 | 4 |
| 354.2405 | 16.938 | -0.03377 | 353.2332 | C_20_H_34_O_5_  C_21_H_32_F_2_O_2_ | 0  9 | 99  1 |
| 366.2769 | 20.580 | -0.02553 | 365.2696 | ND |  |  |
| 366.2771 | 19.219 | -0.01114 | 365.2698 | ND |  |  |
| 392.1114 | 3.857 | -0.03377 | 391.1041 | C_19_H_20_O_9_ | 1 | 4 |
| 420.2509 | 17.570 | -0.02156 | 419.2436 | C_24_H_36_O_6_ | 0 | 2 |
| 422.2675 | 21.278 | -0.02330 | 421.2602 | C_24_H_38_O_6_ | 1 | 11 |
| 424.2823 | 23.071 | -0.03376 | 423.275 | C_24_H_40_O_6_ | 0 | 46 |
| 426.2982 | 11.676 | -0.03377 | 425.2909 | C_20_H_38_N_6_O_4_ | 6 | 1 |
| 428.2049 | 5.985 | -0.03377 | 427.1976 | C_21_H_32_O_9_ | 0 | 1 |
| 428.2051 | 7.697 | -0.03377 | 427.1978 | C_21_H_32_O_9_ | 1 | 1 |
| 428.2053 | 6.511 | -0.03377 | 427.198 | C_21_H_32_O_9_ | 1 | 1 |
| 456.2583 | 6.699 | -0.03377 | 455.251 | C_24_H_40_O_6_S | 8 | 3 |
| 456.2723 | 10.374 | -0.03377 | 455.265 | ND |  |  |
| 492.3565 | 15.158 | -0.03593 | 491.3492 | C_33_H_48_O_3_ | 7 | 2 |
| 492.3569 | 15.772 | -0.03377 | 491.3496 | C_33_H_48_O_3_ | 6 | 2 |
| 494.3716 | 16.218 | -0.03566 | 493.3643 | ND |  |  |
| 506.1789 | 3.426 | -0.03377 | 505.1716 | C_25_H_30_O_11_ | 0 | 2 |
| 506.1792 | 3.937 | -0.03377 | 505.1719 | C_25_H_30_O_11_ | 0 | 2 |
| 515.2961 | 5.874 | -0.03377 | 514.2888 | C_26_H_45_NO_7_S | 8 | 9 |
| 518.3717 | 16.511 | -0.03377 | 517.3644 | ND |  |  |
| 520.3869 | 17.704 | -0.03377 | 519.3796 | C_35_H_52_O_3_ | 9 | 1 |
| 560.0625 | 1.760 | -0.02494 | 559.0552 | ND |  |  |
| 560.116 | 1.544 | -0.00968 | 559.1087 | C_26_H_24_O_14_ | 1 | 2 |
| 590.127 | 1.532 | -0.01742 | 589.1197 | C_34_H_22_O_10_  C_26_H_24_O_15_ | 9  0 | 1  1 |
| 601.1219 | 1.615 | -0.03108 | 600.1146 | ND |  |  |
| 608.136 | 1.527 | -0.02893 | 607.1287 | C_27_H_28_O_16_ | 2 | 8 |
| 612.2995 | 14.870 | -0.03377 | 611.2922 | C_33_H_44_N_2_O_9_ | 8 | 1 |
| 646.3587 | 21.936 | -0.03375 | 645.3514 | C_32_H_54_O_13_ | 3 | 2 |
| 648.3625 | 15.873 | -0.03376 | 647.3552 | ND |  |  |
| 650.3775 | 15.846 | -0.03377 | 649.3702 | C_39_H_54_O_8_ | 6 | 2 |
| 650.378 | 14.101 | -0.03377 | 649.3707 | C_39_H_54_O_8_ | 5 | 2 |
| 666.373 | 16.823 | -0.03376 | 665.3657 | ND |  |  |
| 668.3877 | 16.132 | -0.03376 | 667.3804 | ND |  |  |
| 668.3885 | 16.495 | -0.03376 | 667.3812 | ND |  |  |
| 686.4853 | 20.204 | -0.03376 | 685.478 | C_38_H_71_O_8_P | 4 | 16 |
| 710.0546 | 1.738 | -0.03377 | 709.0473 | ND |  |  |
| 710.1158 | 1.660 | -0.00807 | 709.1085 | ND |  |  |
| 712.2212 | 5.498 | -0.03377 | 711.2139 | C_32_H_40_O_18_ | 0 | 3 |

*ND: Not determined
